# Environmental drivers of forest structure and stem turnover across Venezuelan tropical forests

**DOI:** 10.1371/journal.pone.0198489

**Published:** 2018-06-21

**Authors:** Emilio Vilanova, Hirma Ramírez-Angulo, Armando Torres-Lezama, Gerardo Aymard, Luis Gámez, Cristabel Durán, Lionel Hernández, Rafael Herrera, Geertje van der Heijden, Oliver L. Phillips, Gregory J. Ettl

**Affiliations:** 1 Instituto de Investigaciones para el Desarrollo Forestal (INDEFOR), Universidad de Los Andes, Mérida, Venezuela; 2 School of Environmental and Forest Sciences (SEFS), University of Washington, Seattle, Washington, United of States America; 3 Universidad Experimental de los Llanos Ezequiel Zamora (UNELLEZ), Portuguesa, Venezuela; 4 Institute of Forest Sciences. Faculty of Environment and Natural Resources, University of Freiburg, Freiburg, Germany; 5 Universidad Nacional Experimental de Guayana (UNEG), Bolívar, Venezuela; 6 Centro de Ecología, Instituto Venezolano de Investigaciones Científicas, Caracas, Venezuela; 7 Department of Geography and Regional Research–Geoecology, University of Vienna, Austria; 8 School of Geography, University of Nottingham, Nottingham, United Kingdom; 9 School of Geography, University of Leeds, Leeds, United Kingdom; Chinese Academy of Forestry, CHINA

## Abstract

Using data from 50 long-term permanent plots from across Venezuelan forests in northern South America, we explored large-scale patterns of stem turnover, aboveground biomass (AGB) and woody productivity (AGWP), and the relationships between them and with potential climatic drivers. We used principal component analysis coupled with generalized least squares models to analyze the relationship between climate, forest structure and stem dynamics. Two major axes associated with orthogonal temperature and moisture gradients effectively described more than 90% of the environmental variability in the dataset. Average turnover was 1.91 ± 0.10% year^-1^ with mortality and recruitment being almost identical, and close to average rates for other mature tropical forests. Turnover rates were significantly different among regions (p < 0.001), with the lowland forests in Western alluvial plains being the most dynamic, and Guiana Shield forests showing the lowest turnover rates. We found a weak positive relationship between AGB and AGWP, with Guiana Shield forests having the highest values for both variables (204.8 ± 14.3 Mg C ha^-1^ and 3.27 ± 0.27 Mg C ha^-1^ year^-1^ respectively), but AGB was much more strongly and negatively related to stem turnover. Our data suggest that moisture is a key driver of turnover, with longer dry seasons favoring greater rates of tree turnover and thus lower biomass, having important implications in the context of climate change, given the increases in drought frequency in many tropical forests. Regional variation in AGWP among Venezuelan forests strongly reflects the effects of climate, with greatest woody productivity where both precipitation and temperatures are high. Overall, forests in wet, low elevation sites and with slow turnover stored the greatest amounts of biomass. Although faster stand dynamics are closely associated with lower carbon storage, stem-level turnover rates and woody productivity did not show any correlation, indicating that stem dynamics and carbon dynamics are largely decoupled from one another.

## Introduction

Tropical forests serve as habitats for more than 45,000 tree species [1], and store up to 262 Pg C or 66% of world’s terrestrial biomass [2]. In total, more than 1 billion people, most of them in the tropics, depend on goods and local and regional services provided by forests [3]. Moreover, the whole world benefits from their global climate services, ultimately reflecting the enormous relevance of these ecosystems for species conservation, climate change mitigation and other ecosystem services.

Tropical deforestation remains a serious concern, with more than 2,000 km^2^ year^-1^ lost between 2000–2012 [4], even with a recent decline in deforestation rates in some countries (e.g., [5]). Degradation and deforestation of tropical forests between 2005 and 2010 released between 0.56 and 1.69 Gt C year^−1^ respectively [6], a number that may account for 10–20% of global carbon emissions [7,8]. Yet, forests in the tropics have helped mitigate climate change by sequestering large amounts of carbon. For instance, between 1980 and 2010, net carbon sequestration in mature forests across countries in the Amazon region was estimated to be greater than carbon emissions from land-use change, and except for Venezuela, those from fossil fuels as well [9].

Understanding the factors driving carbon dynamics in tropical forests has become a fundamental task in ecology, management and conservation. The production of standing living biomass is primarily a function of the rate of fixation of CO_2_ by photosynthesis in the forest canopy, and as such is the primary measure of carbon supply and metabolic activity at the individual tree scale [10]. At the stand scale, biomass is the cumulative outcome over time of how environmental factors (e.g., climate, soils) and functional traits (e.g., leaf area and wood density) impact forest structure and dynamics, including the rate at which wood is produced (growth) and lost (branch-fall and mortality) [2].

In highly diverse tropical forests, composed of hundreds or thousands of tree species, each with its own ecological properties, the importance of this diversity on biomass carbon storage is actively debated. Some studies have shown a positive effect of taxonomic diversity on forest carbon (e.g., [11]), but a recent analysis of more than 300 1-ha plots across the tropics found tree diversity and biomass to be largely uncorrelated in Amazonia, Africa, and tropical Asia [12]. Also, while a recent review of empirical studies found that some biodiversity attributes (e.g., species richness) may affect carbon stocks, other vegetation attributes (e.g., community-mean of wood density or specific leaf area) and structural characteristics (e.g., tree density, basal area) appeared more influential [13]. Thus, there is evidence that variables such as wood density [14,15], tree density, and basal area all have some impact on the spatial variation in aboveground biomass of tropical forests [16,17]. Overall, these studies show that stands dominated by medium to high wood density species tend to have higher amounts of biomass, along with lower turnover rates, with water availability being a fundamental limiting factor (e.g., [18,19]). As a result, in the Amazon region, high biomass sites are often in the Central-Eastern and Guiana Shield systems [16,20], including some sites in Southern Venezuela, while low biomass is associated with ‘hyper-dynamic’ forests of southern Amazonia [21].

Other factors may be at play too, and potentially interacting in complex ways. For example, differences in forest biomass and structure across the Amazon Basin were found to be influenced by soil properties and climate [22]. Differences in woody productivity were correlated most strongly with total soil phosphorus, while stem turnover rates were most strongly correlated with a soil physical structure index which combines soil depth, texture, topography and anoxia [22]. An almost two-fold variation in turnover rates between the eastern and western portions of the basin, previously reported by Phillips et al. [23], is associated with low fertility and well-developed soils in eastern, and central Amazonia, versus western Amazonian forests where higher fertility and less-structured substrates predominate. This implies that the processes of stem turnover (i.e., recruitment and mortality) and woody coarse productivity need to be considered to properly understand spatial variation in highly salient structural parameters such as biomass.

Rates of stem turnover have been shown to be well correlated with productivity patterns at global scales in at least two ways: a) through bottom-up relationships (e.g., higher soil fertility inducing faster growth) or b) top-down mechanisms (e.g., higher potential for secondary production) [24]. If high turnover does indeed drive higher productivity, or vice versa, we expect them to be correlated, as has been shown to a limited extent (e.g., [23]). However, whether, and how, these translate into higher biomass is unclear. Most dynamic global vegetation models posit a clear link between the rate of carbon production and the rate of carbon storage, which can be traced back at least to the work of Whittaker and Likens [25], revealing a positive relationship between productivity and biomass in forests of North America. Yet, spatial variation in tropical forest turnover rates is also associated with variations in floristic composition [26,27], and so the relationship between productivity and biomass may not be straight forward. For instance, high turnover rates in high-productivity forests may limit biomass by promoting the dominance of species with a low wood density, and thus an increase in productivity does not necessarily favor increases in forest carbon storage [28]. Furthermore, high productivity values have sometimes been documented at some lowland sites where turnover rates are usually low, including in the Guiana Shield region [29].

**Fig 1:**
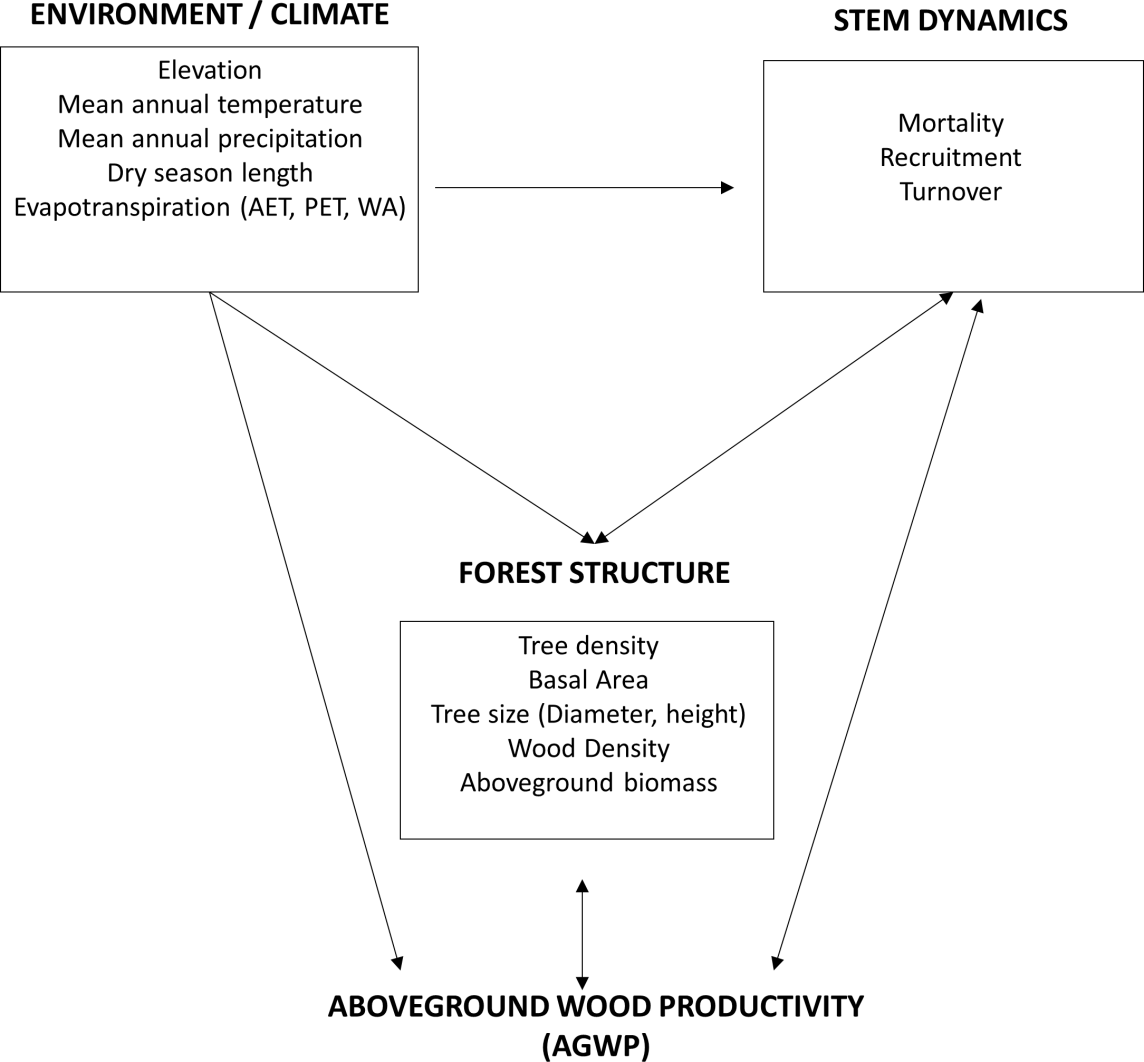
Conceptual model for the analysis of the relationships between turnover rates, aboveground woody productivity (AGWP), and biomass (AGB) including forest structure and environmental descriptors. Bidirectional arrows indicate potential two-way relationships.

Thus, while several empirical and simulation studies have contributed to understanding the process of biomass accumulation and its spatial variation in the tropics [16,22,30,31], it is important to explore how these interactions between environmental factors, turnover rates, productivity and biomass are operating at all scales. In this study, we conduct a comparative analysis of these processes in different forest types across six major bioregions in Venezuela, which has some of the world’s greatest ecological variation of any country in the tropical zone or beyond [32–34]. We build our analytical approach upon previous studies which have analyzed these processes individually or simultaneously mostly at the pan-Amazon scale [17,22,29]. A two-way interaction between stem dynamics and forest structure along with the influence of climatic factors are both key components of our model (Fig 1). An important difference from previous studies is that this work expands the analysis beyond lowland forest sites to include middle to high elevation sites from the Andean biogeographical region, and dry forests sites near the Caribbean Sea in Eastern Venezuela.

Venezuela encompasses a highly diverse set of environmental conditions which are reflected in the diversity of biomes, regions and ecosystems where different forest-types cover about 50% of the land [4], and with approximately 90% of forests located in the Guiana Shield region, south of the Orinoco river [35], the last major forest frontier in Venezuela [36]. In this study, we take advantage of continuous data from 50 permanent plots located in different parts of the country to address three main questions: 1) Are there differences in turnover, aboveground biomass (AGB) and wood productivity (AGWP) among contrasting ecological regions?; 2) To what extent does climate influence structure, stem dynamics and AGB?; and 3) What are the relationships between turnover rates, aboveground biomass and productivity? We first test the general hypothesis that turnover rates differ significantly between the regions and predict that lowland forests with shorter or no dry seasons potentially would have the lowest dynamic rates and would account for the highest values in AGB. A second hypothesis is that environmental conditions (i.e., high annual precipitation and high temperatures) are fundamental drivers of aboveground woody productivity (AGWP), and thus AGB, predicting a positive relationship between these variables. Finally, we consider our results in relation to other studies that have simultaneously addressed turnover, AGB and AGWP, both from field data and/or using remote sensing techniques.

## Materials and methods

### Ethics statement

This study was carried out in strict accordance with correspondent Venezuelan legislation. Every plot census was done after obtaining the required permits in those cases needed for access to protected areas or collecting plant material.

### Permanent sample plots

**Fig 2:**
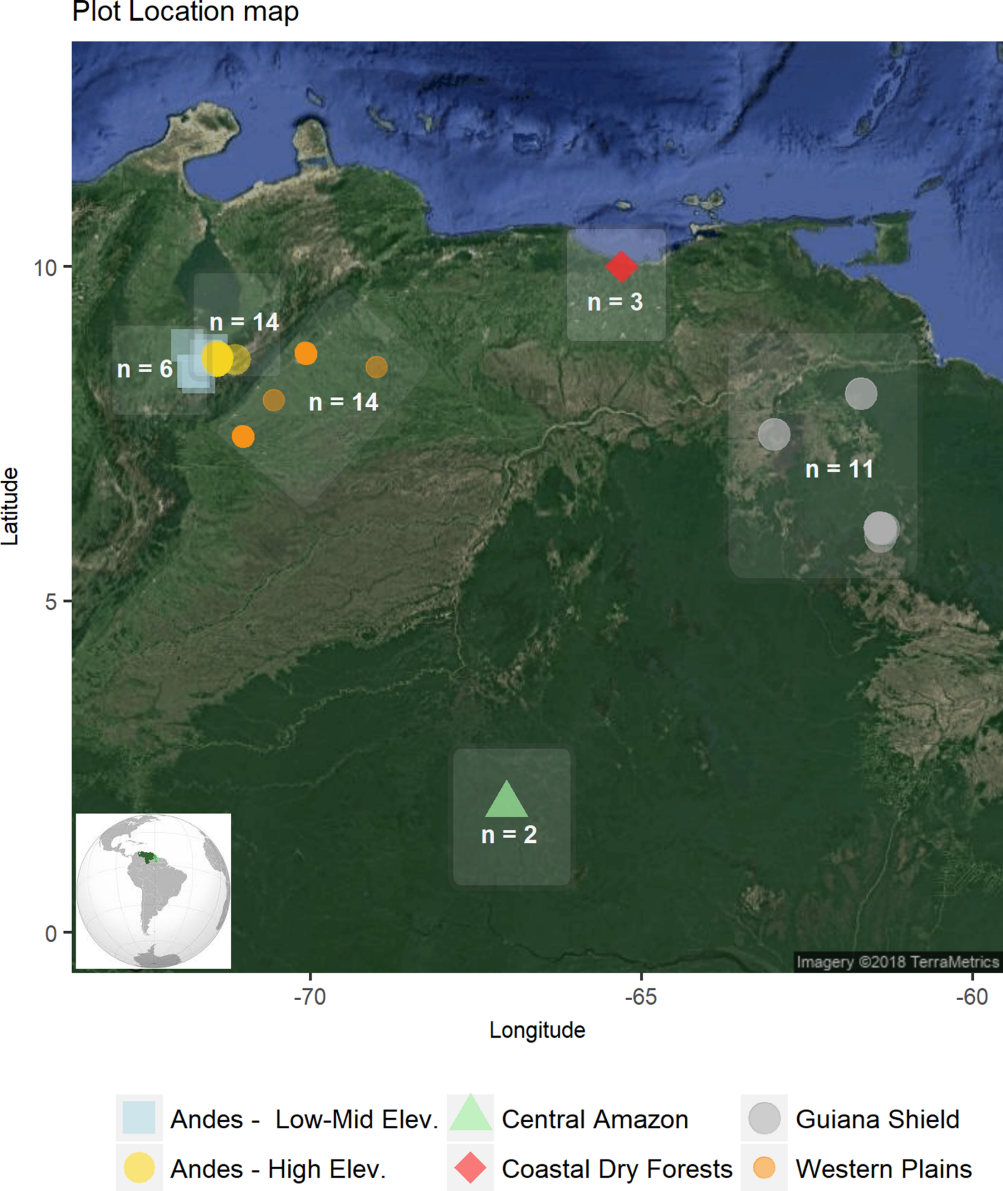
Geographical distribution of forest plots used in this study.

A team of researchers initially led by Jean Pierre Veillon and others thereafter, established in the 1960s and late afterwards a systematic Venezuelan forests plot monitoring network, with some of these plots being the longest running sites in Latin America and the tropics [37–39]. Here, we used data from 50 mature forest plots ranging from 0.1 to 1 ha in area (mean plot size = 0.32 ha), and spanning a wide range of environmental conditions in temperature, rainfall, elevation, disturbance regime, soils, forest structure and species composition (Fig 2). Plots were not equally distributed across biogeographical regions, and thus some areas have been more extensively sampled than others. Temporally, the plots are characterized by a census period ranging from 8 to a maximum of 55 years. First censuses were conducted between 1960 and 2004 (mean year of first census = 1973.8 ± 12.2 Standard Deviation), and final plot censuses between 1972 and 2016 (mean year of last census = 2003.4 ± 15.6). On average, plots were resampled almost 20 times (min = 3 censuses; max = 41 censuses), with an average monitoring period of 29.7 ± 16.43 years (min = 2.0 years; max = 54.8 years) (S1 Table).

Plots were classified into six different regions defined by major climatic conditions, geographical location, and the nature and geological age of the soil substrate as: a) Central-Eastern Amazon (n = 2 plots); b) Andes Low-Mid Elevation (≤ 1.500 meters above sea level—masl) (n = 6); c) Andes High Elevation (> 1500 masl) (n = 14); d) Guiana Shield (n = 11); e) Coastal Dry Forests (n = 3); f) Western Plains (n = 14) (S1 Fig). Geomorphologically, there are clear differences with regards to soil genesis among all sites. Plots located in the Central-Eastern Amazon region correspond to the typical definition of a ´*Terra Firme*´ forest located on hills of Ferrasols covered by a sandy layer, and to a lesser extent by Acrisols and/or Alisols [40]. Soils are well-structured, and due to the presence of patches of macro-porosity and lower bulk density, roots can often penetrate to deeper horizons [41]. Montane forests located in the Andean region are established where soils are shallow and generally less developed (Cambisols) with increasing slope, and with a higher effective depth and development in flat or partially flat terrains, which are also typically enriched with clay (older Cambisols and/or Acrisols/Alisols) [42]. Soils of the Guiana Shield fall into two major groups: Acrisols or Ferrasols [43], typically with low fertility and high iron concentrations. For two of the plots in this region (ELD-3 and ELD-4) soils are mostly shallow with some trees growing directly on rocks that are highly resistant to weathering, with these being classified as Leptosols [41]. Poorly-developed soils are the main feature in the lower montane and dry forests of the eastern coast of Venezuela (plots CLA-03, CLA-04, SAR-03), characterized by clay-loamy textures [44,45]. Finally, the Western Plains region is formed as a Pleistocene-Holocene depositional area of Andean material mostly in the form of alluvial terraces, with microtopographic variations largely determining soil texture and structure [46,47], and with water availability being a limiting factor for plants during dry season [48].

Data collection involves the measurement of all live stems from all species with diameter (D) ≥ 10 cm at 1.3 m height when possible. Standardized protocols have been continuously employed for non-cylindrical stems owing to buttresses or other deformities. In these cases, the point of measurement is raised above the point where stems are more or less cylindrical, approximately 50 cm above the end of the buttress or deformity. The exact height of the point of measurement (POM) has been recorded and marked on the trees to ensure that subsequent measurements are taken at the same point along the stem. In each census, we also accounted for all individuals that had died or were recruited during the interval including possible causes of death [38,49]. Since 2004, all plots are part of the Amazon Forest Inventory Network (RAINFOR) [50,51] where data have been curated and shared via the ForestPlots.net database [52]. Based on the information available for all censuses of each plot we have monitoring data for about 20,400 live stems from 571 identified species from 71 botanical families across all sites. Additional details about field protocols can be found in Brienen et al. [53] and Phillips et al. [49]

### Climatic variables

Climatic data (mean annual temperature and precipitation, and minimum monthly temperature) for each plot was obtained from local weather stations where available. The WorldClim database at 2.5-min or 5-km spatial resolution [54] was used when local information was not available. Since water stress is important in predicting the shape of local allometric equations that are commonly used for estimating aboveground biomass, we also included data on Maximum Climatic Water Deficit (CWD) as defined by Chave et al. [55], which accumulates the monthly differences between monthly rainfall and monthly evapotranspiration. Based on geographical location of each plot, CWD was extracted from a global raster file below 2.5 arc-minute resolution available from http://chave.upstlse.fr/pantropical_allometry.htm (see more in Chave et al. [55], S1 Table). We also included three additional variables related to water availability: estimated actual evapotranspiration (AET; mm year^-1^) and estimated potential evapotranspiration (PET; mm year^-1^), both obtained from the Geospatial Database CGIAR Consortium for Spatial Information [56], and available water (WA), estimated as the difference between mean annual precipitation (MAP) and PET.

Overall, mean annual precipitation is 1,676 ± 799.5 mm year^-1^ (standard deviation) and mean annual temperature 23.03 ± 4.88°C for all plots, with the highest temperature in the Central-Eastern Amazon region and precipitation in the ‘*Sierra de Lema’* zone (SDL plot cluster) of the Guiana Shield (S1 Table and S1 Fig). Plots covered a wide altitudinal range from 50 to 2,450 meters above sea level (mean = 894 ± 952.6 masl). Most sites are characterized by one clear dry season (mean = 3.9 ± 2.1 dry months). Nine sites, mostly in the southern portion of the Guiana Shield and Central-Eastern Amazon were classified as non-seasonal. All climatic variables were included in a Principal Component Analysis (PCA) as this method reduces multivariate data to a smaller number of variables by creating linear combinations of the original variables [57]. Following recommendations from McCune et al. [58] all variables were normalized by range prior to the PCA analysis. These analyses were conducted using the vegan package version 2.4.4 [59] within the R software version 3.4.1 [60].

### Estimation of turnover rates

We estimated demographic rates (% year^-1^) for each plot based on the instantaneous rates approach using the following equations reported in several studies [23,26,29,61]:

$$\text{Annual}\;\text{mortality}\;\left(m\right)=\frac{\ln\left(n_{0}\right)-\ln\left(n_{0}-n_{\text{D}}\right)}{t}x\;100$$

$$\text{Annual}\;\text{recruitment}\;\left(r\right)=\frac{\text{ln}\left(n_{0}-n_{\text{D}}+n_{\text{r}}\right)/\left(n_{0}-n_{\text{D}}\right)}{t}x\;100$$

Where: *n*_0_ is the number of individuals alive at the beginning of the census interval, *n*_D_ is the number of stems that died in the interval, *n*_r_ is the number of individuals recruited between censuses, and *t* corresponds to census interval length. Turnover rate was calculated as the average of recruitment and mortality [23,24]. It has been shown that estimates of demographic rates for heterogeneous populations are influenced by the census interval [23,62]. Therefore, we standardized our estimates of all rates to comparable census intervals using the equation of Lewis et al. [63]: λ_corr_ = λ x t^0.0759^, where λ_corr_ is the rate standardized to a 1-year census interval; λ is the uncorrected demographic rate; *t* is the length of census interval, and 0.0759 is a constant. We calculated corrected values of recruitment, mortality and turnover for each census interval and for each plot in the data set, and calculated average values per plot, weighted by the census interval length.

### Aboveground biomass and woody productivity

Aboveground biomass of each plot was calculated using the moist forest allometric equation from Chave et al. [64]:

$$\text{AGB}=0.0509\times \rho D^{2}H$$

Where AGB is the biomass of each stem (kg), *D* is stem diameter (cm), *ρ* is stem wood density (g cm^-3^) and *H* is stem height (m). The height of each tree was estimated from tree diameter using a height-diameter Weibull equation with different coefficients for each region [65]. Following Baker et al. [14], the wood density of each tree was assigned on a taxonomic basis from the pan-tropical database of Zanne et al. [66], first by species, and when this was not available we used data at the genus and family levels, while mean plot-level wood density values were used when taxonomic information was missing. We assumed carbon to be 50% of total dry biomass, and as suggested by Malhi et al. [16] and Johnson et al. [29] we added an additional 6.2% of carbon to each AGB-plot estimate to account for the unmeasured small trees (<10 cm in diameter).

For each census interval, above-ground wood productivity (AGWP) was estimated as the sum of AGB gains of surviving and recruiting trees, with AGB mortality as the summed AGB of trees dying over the interval. AGWP and AGB mortality were corrected to include two small unobserved components relating to trees that die within the census interval: (1) biomass gain and loss of the cohort of unobserved recruits that both enter and die between two successive censuses, and (2) unobserved biomass gain and loss of known trees that die between two successive censuses. To correct for this, we followed the empirical procedure proposed by Talbot et al. [67]:

$${\text{AGWP}}_{\text{corr}}={\text{AGWP}}_{\text{obs}}\times 0.0091{\text{AGWP}}_{\text{obs}}\times t$$

Where AGWP_obs_ is the uncorrected value of woody productivity, and *t* is the length of census interval. Since our census intervals were frequent (mean number of censuses = 20, with an average of 29.6 years of total monitoring period), the effect of this adjustment is minor. Finally, to compute comparable estimates of AGWP, we calculated corrected values of every AGB component for each census interval and for each plot, and calculated average values per plot weighted by census interval length.

### Statistical analysis

General statistics for all turnover rates, AGB and AGWP were calculated (i.e., mean, standard error) on a per plot basis and by region. We allocated all plots to three seasonality conditions: Aseasonal (0–1 months with less than 100 mm in precipitation); Slightly seasonal (2–3 dry months); Seasonal (> 3 dry months). Following Phillips et al. [23], plots were allocated to two major soil fertility classes, broadly defined by published soil profiles from each region and data from a limited number of plots [41] (S1 Table). Estimates of recruitment and turnover rates were both normally distributed (Recruitment: *W* = 0.975, p = 0.398, Shapiro-Wilk normality tests; Turnover: *W* = 0.965, p = 0.137), while mortality rates were not (*W* = 0.926, p = 0.004). Thus, for recruitment and turnover we used an analysis of variance (ANOVA) to test for statistical differences among each region, seasonality condition and soil fertility, while a Kruskall-Wallis test was used for mortality. Similarly, a parametric post-hoc Tukey test was conducted for recruitment and turnover when differences were found, and a non-parametric Dunn-test in the case of mortality. Estimates of AGB and AGWP were normally distributed (AGB: *W* = 0.987, p = 0.863; AGWP: *W* = 0.972, p = 0.282), while biomass loss (AGB_mort_) was not (*W* = 0.905, p < 0.001). To test for regional differences of AGB, AGWP and other AGB components in Venezuelan forest plots we followed the same approach as set out for turnover rates above.

To explore the question of potential drivers of turnover rates, biomass and productivity, we followed four analytical steps. First, we applied individual simple correlations using Kendal’s *τ* (tau) approach to examine overall trends between all variables as it does not rely on a particular distribution of the variables involved (S2 Table). Second, we tested the relationships between our three response variables (turnover, AGB and AGWP) with environmental descriptors using the scores of the first two axes obtained from the Principal Component Analysis (PCA) explained earlier. Third, we used simple linear models to explore how turnover, AGB, and AGWP are affected by each one of the explanatory variables classified by group (i.e., climate, dynamics, and structure). To account for all potential correlations we also included turnover rates as an explanatory variable of both AGB and AGWP. The effects of every AGB component on the total AGB including biomass and productivity were also included. Finally, we used generalized least squares (GLS) regression models to further explore these relationships, where we accounted for the spatial autocorrelation across the plot network by specifying a Gaussian spatial correlation structure. We conducted our GLS regressions using prior information from the linear modeling and literature (e.g., [19,23,29,61]), and models were tested based on two groups of parameters (climate and structure). The explanatory variables were standardized by fitting a mean equal to 0 and variance to 1 to directly compare the effects of all variables. Model selection was based on the corrected version of the Akaike information criterion (AICc) to correct for small sample size [68] combined with a k-fold cross-validation method [69]. This approach allows the performance of models to be assessed randomly dividing the data into k groups (k = 10 in this study) and the model is adjusted k times, so in each run one of the k groups is used as a test set. Prediction error was calculated as the relative difference in the root mean squared error (RMSE) between the full version of the models and the training models. We conducted these analyses using the “scale” and “gls” functions from the nmle package [70] and “AICc” function from the MuMIn package [71], all in the R software version 3.4.1 [60].

## Results

### Environmental variability

**Fig 3:**
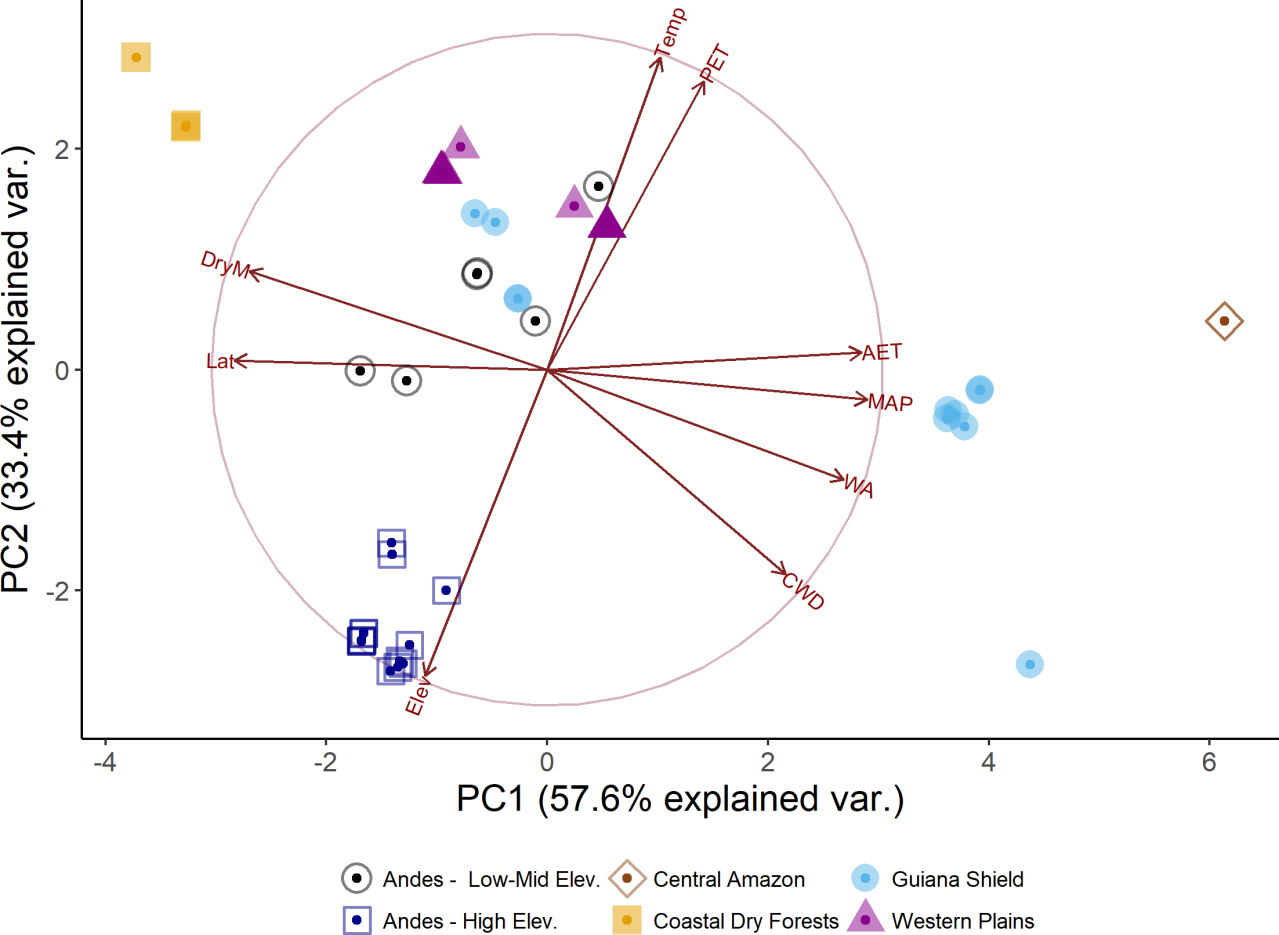
Principal component analysis (PCA) on nine environmental variables of all plots and bioregion. PCA axis 1 largely represents decreasing moisture supply, while axis 2 is mostly associated with increasing temperatures. See correlations between PCA variables in S2 Table and S2 Fig.

Correlations between environmental factors were mixed. Not surprisingly, some of the highest values were found for actual evapotranspiration (AET) and annual precipitation (MAP) (Kendal’s tau τ = 0.80), and elevation and temperature (τ = - 0.78) (S2 Table and S2 Fig). The Principal Component Analysis shows two major axes which explained more than 90% of the environmental variability in the dataset (Fig 3). A first axis captured 57.6% and was negatively correlated with latitude, elevation and length of dry season (DryM), and positively correlated with annual temperature (MAT), annual precipitation (MAP), actual and potential evapotranspiration (AET, PET), available water (WA) and Climatic Water Deficit (CWD). The second axis described 33.4% of the variation with elevation, MAP, WA, and CWD negatively correlated, and positively correlated for Latitude, MAT, AET, PET, and length of dry season (Table 1).

**Table 1:** Loadings and variation explained by three major axes of variation using nine environmental variables recorded for 50 permanent plots in Venezuelan forests. In bold the variables with loadings above 0.3 for each PCA Axis. PCA axis 1 largely represents decreasing moisture supply, while axis 2 is mostly associated with increasing temperatures.

PCA variable	Comp.1	Comp.2	Comp.3
Latitude	**- 0.409**	0.016	0.220
Elevation	- 0.160	**- 0.526**	- 0.152
Mean Annual Temperature (MAT)	0.148	**0.535**	0.055
Mean Annual Precipitation (MAP)	**0.419**	- 0.052	**0.463**
Actual Evapotranspiration (AET)	**0.411**	0.029	-0.455
Potential Evapotranspiration (PET)	0.205	**0.495**	-0.218
Available Water (WA)	**0.388**	- 0.189	**0.549**
Number of Dry Months (DryM)	**- 0.389**	0.169	-0.012
Climatic Water Deficit (CWD)	**0.311**	**- 0.351**	-0.394
Standard Deviation	2.277	1.733	0.590
Proportion of Variance	0.576	0.334	0.037
Cumulative Proportion	0.576	0.910	0.948

### Turnover rates, biomass and productivity

**Fig 4:**
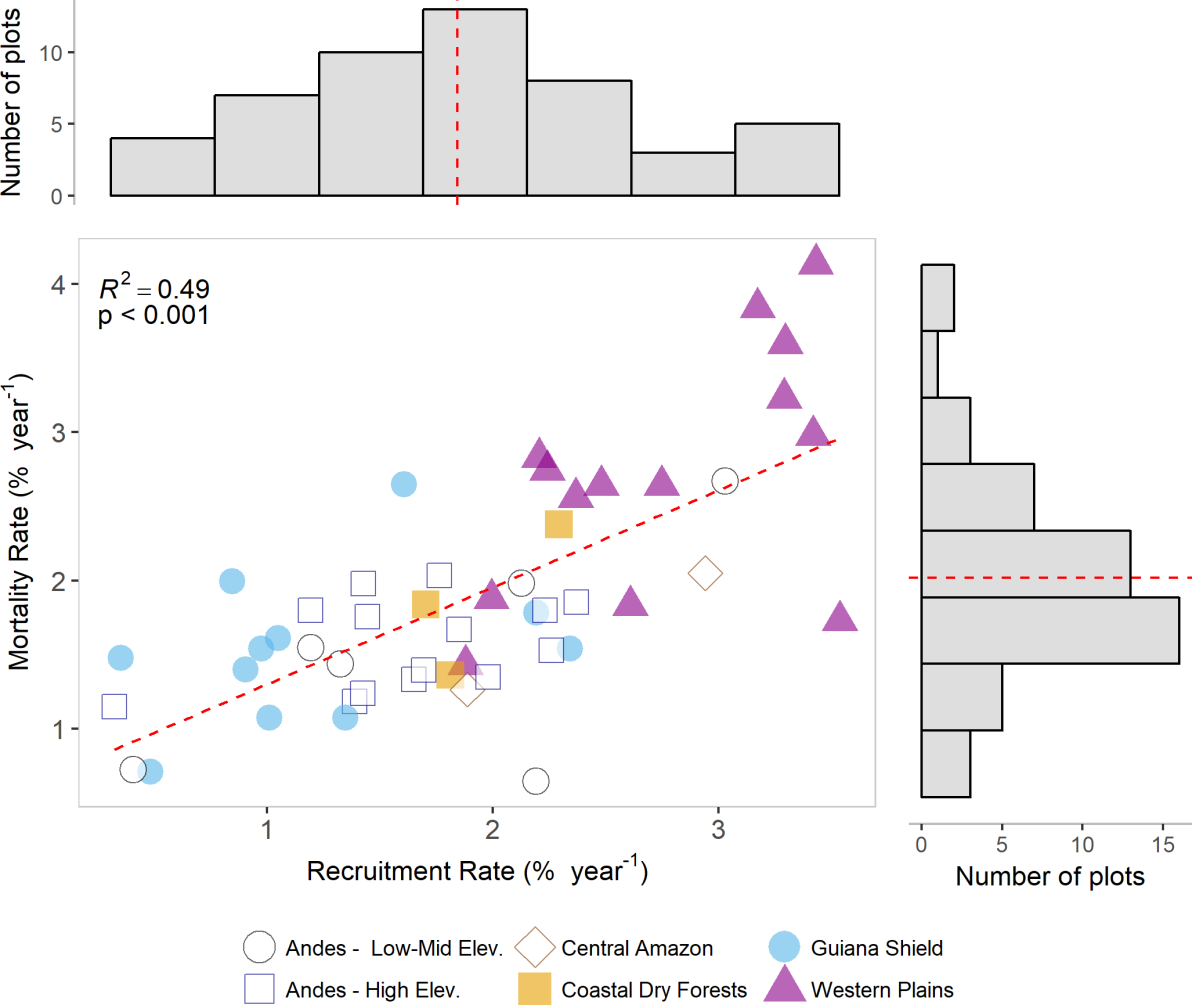
General distribution of mortality and recruitment rates classified by region. Red line in the histograms represent the average for both demographic rates. Red line in the scatter plot is a linear fit between mortality and recruitment.

Average estimates of turnover rates were 1.91 ± 0.10% year^-1^ (*r* = 1.91 ± 0.12; *m* = 1.89 ± 0.11 –Standard Error of the Mean) for all plots, and mortality rates and recruitment rates are positively correlated (Fig 4 and S3 Table). The maximum regional turnover is 2.74 ± 0.17 and the minimum of 1.36 ± 0.14% y^-1^ for the Western Plains and Guiana Shield regions respectively. In most regions mortality and recruitment rates are balanced (i.e., close to the fitted line); however, some Western Plains sites showed mortality exceeding recruitment (S3 Table). The plots averaged 159.4 ± 7.3 Mg C ha^-1^ in AGB, and varied regionally between 100.6 ± 26.5 Mg C ha^-1^ for Coastal Dry Forests, and 204.8 ± 14.3 Mg C ha^-1^ in the Guiana Shield. Similarly, Guiana Shield forests were the most productive with an average carbon uptake in the woody biomass (AGWP) of 3.26 ± 0.28 Mg C ha^-1^ y^-1^, while AGWP was lowest in the drier areas of the eastern coast of Venezuela, with an average of 1.77 ± 0.48 Mg C ha^-1^ y^-1^ (Table 2).

**Table 2:** Summary results of three groups of forest metrics for six major bioregions in Venezuelan forests. Values are means ± standard errors. Tests for differences among regions are provided. In bold the highest value for each variable.

Forest Property / Bioregion		All regions (n = 50)	Andes Low-Mid Elevation (n = 6)	Andes—High Elevation (n = 14)	Central Eastern Amazon (n = 2) ^a^	Coastal Dry Forests (n = 3)	Guiana Shield (n = 11)	Western Plains (n = 14)	Statistic ^b^	*p*
**Forest structure**	Basal Area (m^2^ ha^-1^)	28.65 ± 1.09	26.87 ± 3.05	**36.17 ± 1.38**	28.68	19.38 ± 5.08	28.50 ± 2.06	23.99 ± 1.37	F = 7.614	< 0.001
	Stem Density (ha^-1^)	529.41 ± 34.53	428.25 ± 61.12	**731.5 ± 48.27**	656.75	482 ± 87.46	625.77 ± 79.44	286.92 ± 13.83	χ^2^ = 32.021	< 0.001
	Plot Wood Density (g cm^-3^)	0.61 ± 0.01	0.59 ± 0.02	0.58 ± 0.01	0.68	**0.73 ± 0.01**	0.69 ± 0.02	0.57 ± 0.01	χ^2^ = 26.69	< 0.001
**Forest dynamics**	Observed Recruitment Rate (% y^-1^)	1.913 ± 0.12	1.711 ± 0.38	1.642 ± 0.14	2.413	1.936 ± 0.18	1.191 ± 0.19	**2.763 ± 0.16**	F = 9.088	< 0.001
	Observed Mortality Rate (% y^-1^)	1.899 ± 0.11	1.503 ± 0.31	1.577 ± 0.08	1.657	1.861 ± 0.29	1.533 ±0.16	**2.721 ± 0.22**	χ^2^ = 18.01	0.003
	Observed Turnover Rate (% y^-1^)	1.906 ± 0.10	1.607 ± 0.31	1.610 ± 0.09	2.035	1.898 ± 0.23	1.362 ± 0.14	**2.742 ± 0.17**	F = 10.39	< 0.001
**Biomass components**	Aboveground Biomass (Mg C ha^-1^)	159.39 ± 7.29	144.89 ± 25.37	173.98 ± 8.00	192.29	100.57 ± 26.47	**204.78 ± 14.28**	123.24 ± 9.00	F = 7.008	< 0.001
	Aboveground Biomass losses—AGB loss (Mg C ha^-1^ y^-1^)	2.20 ± 0.18	1.52 ± 0.36	2.20 ± 0.17	2.62	0.79 ± 0.13	**3.47 ± 0.53**	1.73 ± 0.21	χ^2^ = 16.786	< 0.001
	Aboveground Woody Productivity (Mg C ha^-1^ y^-1^)	2.73 ± 0.11	2.94 ± 0.21	2.39 ± 0.14	2.75	1.76 ± 0.48	**3.26 ± 0.28**	2.77 ± 0.18	F = 3.284	0.0132

^a^ Not possible to calculate standard error due to low sample size (n = 2).

^b^ ANOVA for normally distributed data, and non-parametric Kruskall-Wallis χ^2^ for other distributions.

**Fig 5:**
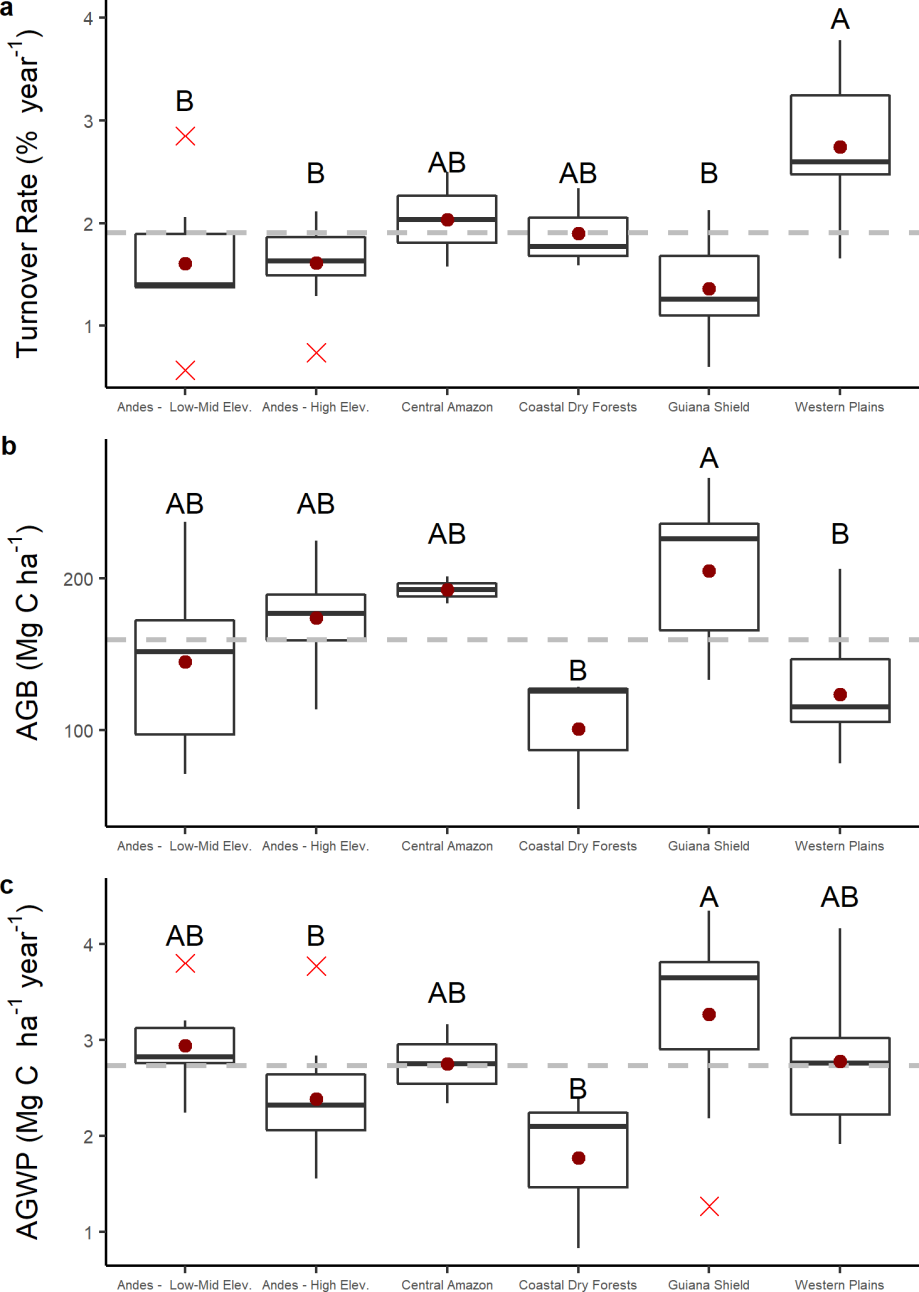
**Boxplots of A) turnover rates; B) Aboveground biomass; and C) Wood productivity, including the results of pos-hoc grouping tests by region. Red dots indicate the average for each variable in each region.** Gray dashed line is the overall mean for each variable. Statistical significant differences were found for turnover (F_44,5_ = 10.39; p < 0.001), AGB (F_44,5_ = 7.008; p < 0.001), and AGWP (F_44,5_ = 3.284; p < 0.05).

ANOVA and Kruskal-Wallis tests showed significant statistical differences for all three response variables (Turnover, AGB, AGWP) among the six regions (Table 2). Post-hoc tests were useful to compare the differences among regions. For instance, the Western Plains had significantly higher turnover than the other regions, while the Guiana Shield has significantly greater AGB and AGWP, while also being in the grouping of lowest turnover rates, with the Western Plains in the lowest group of AGB (Fig 5).

Climate seasonality and soil fertility were associated with significant differences in turnover rates, AGB, and, to a lesser extent, AGWP. Turnover rates were different among the three categories of seasonality (F = 4.669, p = 0.014) with highly seasonal sites (> 3 dry months per year) being the most dynamic. These differences are mostly driven by recruitment; although we found higher rates of mortality for these group of plots, a non-parametric test showed no differences in mortality with seasonality (χ^2^ = 3.302; p = 0.19). Both AGB and AGWP showed the same pattern with decreasing AGB and AGWP with increasing seasonality (aseasonal sites > moderately seasonal sites > highly seasonal sites). Turnover rates were higher for the high fertility plot group (F = 13.19, p < 0.001). Conversely, AGB was higher in the low fertility sites (F = 8.933, p = 0.004). AGWP was greater among the high soil fertility plots (n = 22, mostly in Western Plains and low-mid elevation forests in the Andes), but not significantly so when compared with the low fertility group (n = 28) (F_48,1_ = 1.102 p = 0.299) (S3 Fig).

### Potential drivers of turnover rates, biomass and aboveground woody productivity

#### Turnover rates

**Fig 6:**
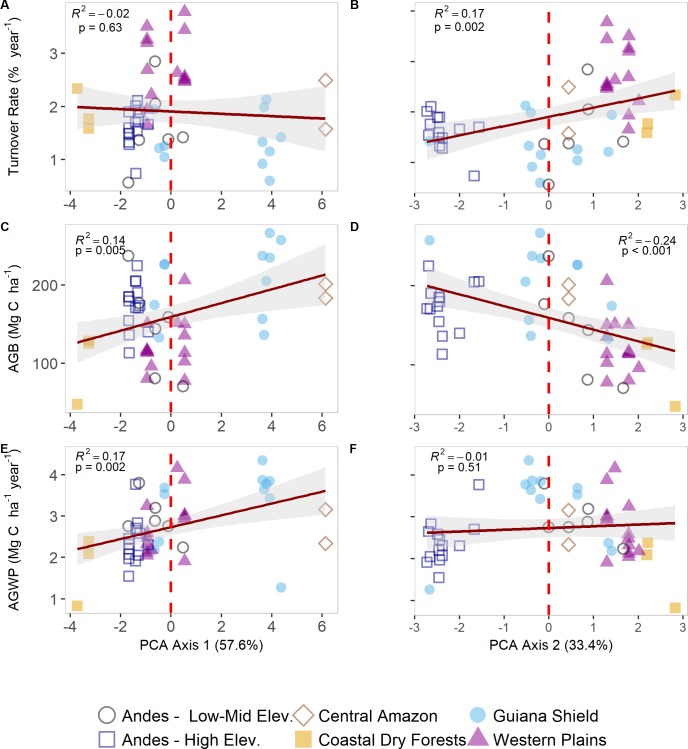
**Relationships between turnover rates (A-B), aboveground biomass (C-D), and aboveground woody productivity (E-F) with two PCA axes by region.** Red dashed line indicates the “zero” value for the scores in each axis. For details on direction of the vectors and loadings see Fig 3 and Table 1.

Turnover rates were significantly positively correlated with the length of dry season, mean annual temperature, climatic water deficit, and potential evapotranspiration, but negatively with elevation. Turnover rates were uncorrelated with the scores of PCA1 axis, but positively correlated with the second PCA axis (R^2^ = 0.17; p = 0.002). Aboveground biomass, basal area, stem density, and stand wood density were structural parameters negatively correlated with turnover (Table 3). Lower rates of turnover, including both recruitment and mortality, were found in sites characterized either by higher elevation and lower temperatures (i.e., Andean forests), or lowland forests with higher water availability from precipitation (i.e., Central Amazon and Guiana Shield) (Fig 6A and 6B).

**Table 3:** General correlations (R^2^) between turnover rates, aboveground biomass and woody productivity and three groups of explanatory variables. Numbers in bold highlight the significant correlations, with numbers in parenthesis indicating the p-value.

Explanatory variables		Response variables		
		Turnover rate	AGB	AGWP
**Environmental**	PCA 1	- 0.02 (0.64)	**0.14 (0.005)**	**0.17 (0.002)**
	PCA 2	**0.17 (0.002)**	**- 0.24 (<0.001)**	- 0.02 (0.51)
	Latitude (Lat)	- 0.02 (0.76)	**- 0.13 (0.007)**	**- 0.08 (0.03)**
	Elevation (Elev)	**- 0.10 (0.01)**	**0.06 (0.05)**	**- 0.05 (0.05)**
	Mean Annual Temperature (MAT)	**0.10 (0.01)**	**- 0.09 (0.02)**	**0.06 (0.05)**
	Mean Annual Precipitation (MAP)	- 0.01 (0.64)	**0.15 (0.003)**	**0.06 (0.04)**
	Number of Dry Months (DryM)	**0.11 (0.01)**	**- 0.35 (<0.001)**	**- 0.22 (<0.001)**
	Actual Evapotranspiration (AET)	- 0.02 (0.74)	**- 0.06 (0.05)**	**0.19 (0.001)**
	Potential Evapotranspiration (PET)	**0.09 (0.02)**	**- 0.05 (0.05)**	0.05 (0.06)
	Available Water (WA)	- 0.01 (0.27)	**0.24 (<0.001)**	0.04 (0.09)
	Climatic Water Deficit (CWD)	**0.09 (0.02)**	**0.30 (<0.001)**	**0.11 (0.01)**
**Stem Dynamics**	Turnover rate	—-	**- 0.37 (<0.001)**	- 0.01 (0.79)
	Recruitment rate	**0.86 (<0.001)**	**- 0.44 (<0.001)**	- 0.02 (0.52)
	Mortality rate	**0.83 (<0.001)**	**- 0.19 (<0.001)**	- 0.02 (0.84)
**Forest** **structure**	Average Stem Density	**- 0.16 (0.002)**	**0.12 (0.007)**	**- 0.12 (0.008)**
	Average Basal Area	**- 0.19 (< 0.001)**	**0.57 (< 0.001)**	**- 0.01 (0.42)**
	Mean Plot Wood Density	**- 0.15 (0.003)**	**0.09 (0.019)**	0.01 (0.41)
	Aboveground biomass (AGB)	**- 0.36 (< 0.001)**	—-	**0.10 (0.015)**
	AGB losses (AGB Mort)	- 0.01 (0.53)	**0.17 (0.002)**	0.01 (0.19)
	Woody productivity (AGWP)	- 0.01 (0.79)	**0.10 (0.015)**	—-

We tested 13 different GLS models based on climatic parameters, and 10 additional models using forest structure as explanatory variables for turnover rates (S4 Table). The best climate-based model (i.e., lowest AICc) describing turnover included the effects of mean annual temperature (MAT) with a regional interaction. Nevertheless, the difference in the log-likelihood and AICc among the first six models was relatively small with potential evapotranspiration (PET), length of dry season and climatic water deficit (CWD) following in the relative importance as climatic drivers of turnover rates. Prediction errors in all models (i.e., the relative difference between the RMSE from the 10-fold validation and the RMSE from the selected model including all data) ranged between 0.2 and 16.5%, with 11.7% in the case of the selected model (Pseudo *r*^*2*^ = 0.55; AICc = 100.57). The average of plot basal area in combination with region were the two structure-based terms composing the best model to explain turnover rates in our dataset. Prediction errors for this group of models ranged from 1.4 to 19.9%. In both cases, the selected models showed a relatively good fit when comparing predicted vs. estimated values of turnover rates while also showing the estimated regional differences in turnover rates as well (S4 Fig).

### Aboveground biomass (AGB)

AGB was negatively correlated with the length of dry season (R^2^ = - 0.35, p <0.001), and positively with available water and mean annual precipitation. The negative nature of CWD also implied a positive relationship with AGB with less negative values (e.g., Central Eastern Amazon) accounting for high biomass. There was also a positive correlation to PCA1 (increasing MAP, AET, WA and CWD) (R^2^ = 0.14, p = 0.005), and a negative correlation with PCA2 (increasing MAT and PET and decreasing elevation). This negative relationship between the scores of PCA2 and AGB (R^2^ = - 0.24, p <0.001) suggests that sites with high turnover rates (e.g., Western Plains) have lower AGB and positive scores on the PCA2 axis (Fig 6D). Andean forests in general, along with Central Amazon and Guiana Shield comprised the sites with the highest aboveground biomass. All demographics rates were significantly negatively correlated with biomass. All forest structure explanatory variables (i.e., basal area, stem density, plot wood density, aboveground biomass losses from mortality, and woody productivity) were positively correlated with AGB (Table 3).

A combination of water availability (WA), CWD, potential evapotranspiration (PET), and region formed the best climate model explaining AGB. The next best single-parameter models showed the relative importance of CWD, PET, length of dry season and WA. Prediction error for all climatic models ranged between 1.8 and 13.9%. Turnover rates, stem density, average wood density, biomass loss and woody productivity were all part of the best structural-based model explaining AGB in our dataset. As in the case of turnover rates, the relationship between the predicted and estimated values was acceptable with a good coefficient of determination (0.51 for the best climate model and 0.63 for the structure-based model), while partially capturing the regional differences among the dataset (S4 Table and S4 Fig).

### Aboveground woody productivity (AGWP)

**Fig 7:**
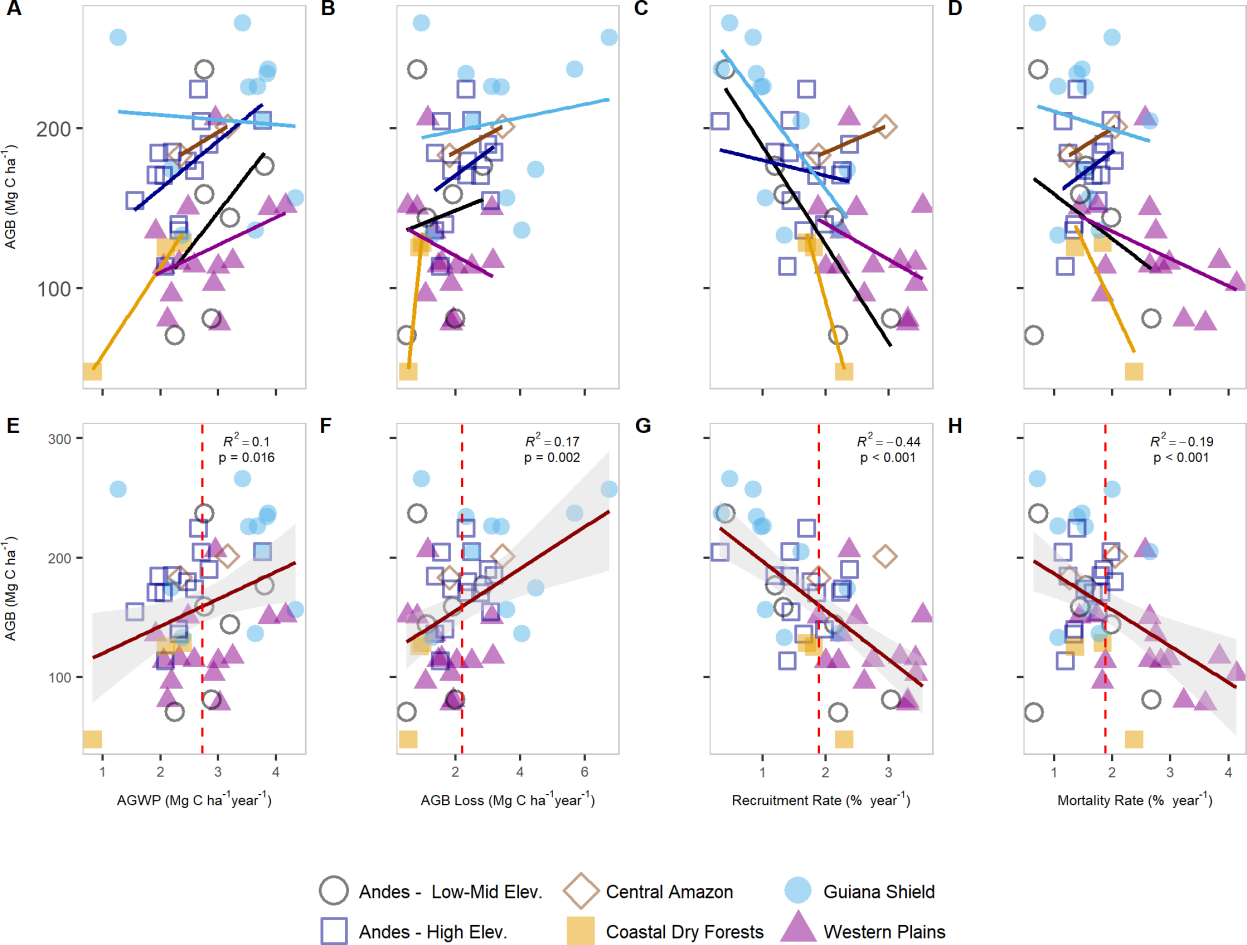
**AGB relationships with AGWP (A & E), AGB loss (B & F), recruitment rate (C & G), and mortality rate (D & H).** Upper panel depicts linear fits for every region. Bottom panels represents a linear fit for all data combined. Red dashed lines are the arithmetic mean for each variable.

AGWP was negatively correlated with the number of dry months, increasing elevation and latitude, and positively correlated with AET, CWD, MAT, MAP, PET, and WA (Table 3). AGWP was significantly and positively correlated with the first PCA axis (R^2^ = 0.17; p = 0.002) and uncorrelated with the second PCA axis (Fig 6E and 6F). Turnover rates were uncorrelated with AGWP; however, stem density was negatively correlated with AGWP. Most forest structure variables were not correlated with AGWP, but there was a positive correlation with AGB (Table 3 and Fig 7).

Using climate variables as predictors, we found that the best model describing AGWP included AET interacting with region, followed by length of dry season, and CWD. Average prediction errors for this group of models was 6.7% (min = 1.2; max = 11.6). GLS modeling confirmed the lack of relationship between most structure variables and AGWP, with only stem density slightly interacting with region as the terms of the best model describing woody productivity. However, the relationship between the predicted and estimated values was poor (S4 Table and S4 Fig).

## Discussion

Across Venezuelan forests, and encompassing a wide environmental gradient we quantified tree turnover rates, aboveground biomass (AGB) and woody productivity (AGWP). Our results reveal significant differences in stand and regional-level patterns of all variables for a broad range of forest habitat types in Venezuela (Figs 4 and 5).

First, PCA was useful in describing relevant environmental differences at the regional level and reflects the overall gradient captured by this dataset. Two orthogonal climatic axes describe two major gradients in moisture and temperature (Fig 3). Length of dry season and water availability differentiate the Central Amazon and Guiana Shield from other regions, with both characterized by the least negative values of CWD and thus highest water availability. At the other extreme of this gradient, we found the most negative values of CWD in the dry forests of the eastern coast of Venezuela. Lowland forests, both at the Guiana Shield and Western Plains were mostly defined by a warmer climate and medium to high levels of precipitation. As expected, lower temperatures at medium to high elevation characterize the position for most of the Andean sites along the ordination space (Fig 3 and S1 Table and S2 Fig).

Secondly, in terms of stem dynamics, mean turnover rates (1.91 ± 0.10% year^-1^) closely matched previous studies that used a sub-sample of our plots and over a much shorter monitoring period [23,42,72]. Turnover rates vary substantially between sites and regions (Fig 4), suggesting that larger samples for all regions may be needed to better distinguish large-scale patterns. However, despite the inherent noise in recruitment and mortality processes, taken together our results further reveal that tree turnover in mature seasonal forests located in the alluvial plains in western Venezuela (2.74 ± 0.17% year^-1^) is not only faster than other areas in Venezuela (mean of 1.82 ± 0.17% year^-1^), but also than in forests of the northern central Andes of South America (e.g., 1.88 ± 0.11% year^-1^, [61]). For western forests in Venezuela, mean turnover rates are close to the range reported for other western Amazonia lowland forests (e.g., 2.49 ± 0.12% year^-1^, [23]). Sites in the Guiana Shield region, however, had the overall lowest turnover rates (1.36 ± 0.14% year^-1^), with average mortality rates (1.53 ± 0.16% year^-1^) being close to other estimates from the region (e.g., 1.66 ± 0.16% year^-1^, [29]) (Figs 4 and 5A).

These regional differences in stem dynamics might indicate a combined effect of prevailing climate, soil conditions and forest structure, hence revealing complex regional patterns in turnover, AGB and AWGP that we have partially unveiled with this dataset. Although having low but significant correlations, the environmental conditions most strongly associated with turnover rates were mean annual temperature (MAT), length of dry season, and to a lesser extent other moisture-related parameters such as potential evapotranspiration (PET) and climatic water deficit (CWD) (Tables 3 and S4). When these variables were grouped in the scores of the second PCA axis we found a significant correlation with turnover rates (Fig 6B). Overall, sites with high dynamism tend to have a higher moisture deficit, which may shed light on the effects of water limitation not only on recruitment and mortality, but also on biomass and productivity.

Previous studies have shown that higher temperatures coupled with stronger water deficit, whether in the form of low precipitation or extended dry seasons or both, are well-known drivers of tree mortality [73–75]. Our results corroborate other studies (e.g., [29,76]) and show that the highest rates of tree mortality were found in the two regions with the strongest water deficit, namely coastal dry forests in Eastern Venezuela and Western Plains (Table 2). The case of recruitment is less clear since sites with higher water availability (e.g., Central Amazon) also had high recruitment rates (mean of 2.41% year^-1^). For instance, in Phillips et al. [23], and using data from 97 sites across the Amazon, a mean recruitment rate of 2.41 ± 0.15% year^-1^ was found for plots classified as non-seasonal. In our study, we found significant differences for recruitment (F = 5.271, p = 0.009) but none for mortality (χ^2^ = 3.85, p = 0.15) when plots were classified according to seasonality, although highly seasonal sites had the highest mortality rate (2.05 ± 0.13% year^-1^) (S3 Fig).

Estimates of AGB are well within the values reported in other regional studies [16,20,29] with the Guiana Shield region accounting for the most carbon-rich forests in Venezuela and the Amazon (204.78 ± 14.28 Mg C ha^-1^), and dry forests in Eastern Venezuela having the lowest values in AGB (100.57 ± 26.47 Mg C ha^-1^) (Fig 5B). Among our three response variables, AGB was the only one significantly correlated with both PC1 and PC2. Biomass increases with increasing water availability (WA) but decreases with an increase in temperature and PET, suggesting that while water deficit may promote higher turnover rates (Fig 6B), it may limit the amount of carbon stored in these forests (Fig 6D). Moreover, in our dataset, WA, CWD and PET were the best environmental predictors of AGB by means of linear and GLS modeling (Tables 3 and S4), with moderately and highly seasonal plots accounting for lower AGB values (162.84 ± 28.08 and 145.63 ± 7.43 Mg C ha^-1^ respectively) (S3 Fig).

Two separate assessments conducted at the pan-Amazon scale [17,22] similarly found strong effects of dry season length on the specific stand characteristics that explained the spatial variation in AGB across different forest types with an east to west gradient in turnover, AGB, and woody productivity (AGWP). These studies also indicated that wet and warm sites support higher biomass forests, which were predominantly composed of high wood density species (e.g., Guiana Shield). Contrastingly, seasonal sites sustained forests with lower tree density, basal area and therefore lower AGB (e.g., Western Plains). There was, however, an important combined effect of soil structure and fertility on turnover and AGB in Quesada et al. [22] in which less weathered soils may promote higher dynamism through soils with less effective-depth, and higher AGWP by a higher phosphorous content. These areas are mostly in western Amazonia where AGB is frequently lower as has been shown previously (e.g., [16,29]).

In our study, the lack of good quality standardized data limited our ability to fully test for the effects of soils in the response variables. However, using a simple approach we were able to allocate all 50 plots into two major soil fertility classes (S1 Table), and found that turnover rates were indeed faster for the high fertility group (n = 22), while AGB was significantly higher for the low fertility group (n = 28). AGWP was also higher in the high fertility group but not significantly so (S3 Fig). These results are consistent with studies showing that forests in tropical Amazonia growing on more fertile soils tend to have higher turnover rates than those with lower fertility, while also being more productive [15,23].

Both recruitment and mortality rates were strongly and negatively correlated with AGB (Table 3 and Fig 7), showing that tropical forests characterized with a high stem mortality risk, mostly by environmental conditions that includes extremes of temperature or longer dry seasons, such as those represented by the plots in the Western plains and the dry coast of Venezuela, tend to support lower biomass [18,19]. Although the relationship between mortality rate and AGB is somewhat weaker than that between recruitment and AGB, it is consistently negative across all sites, and within most of the regions with the strongest gradients in mortality (Fig 7). This supports recent findings showing that stem mortality rates determine spatial variation in AGB in the Amazon [29,77]. However, contrary to the results from Johnson et al. [29] where no correlation between AGB_mort_ and AGB was found, we see a positive relationship within our plots, matching the rather weak but significant relationship between AGB and woody productivity (Fig 7). Since our most productive plots (i.e., Guiana Shield) also had the highest estimates of both total AGB and AGB_mort_, we would probably need a larger sample size to confirm whether the saturation effect in the AGB-AGWP relationship reported in Keeling and Phillips [28] also holds here.

The relationship between turnover and AGB is an example of how stem dynamics can drive biomass accumulation, thus different mechanisms of tree mortality may affect forest structure, which in turn may affect forest biomass. Observations on mode of mortality in our plots indicate that the high mortality rates of some plots (e.g., Western Plains) are driven by more dynamic death events (e.g., broken and/or uprooted trees, and often involving more than one individual). In less dense forests, trees with low to medium wood density are more exposed to strong wind disturbances and are more likely to die because of stem breakage or by being uprooted, likely associated with soil physical conditions as shown by Quesada et al [22]. These particular modes of death are often associated with the creation of larger canopy gaps compared to those created by trees that die standing. In northern Amazonia, both growth rate and wood density were found to be good predictors of tree mortality and modes of death [78,79], with mortality probabilities depending both on physiological failure (e.g., drought) and mechanical failure (small size, slow growth and mode of breakage). However, in our dataset, the drier plots in eastern Venezuela, which had a high average plot wood density (S3 Table), also had a high overall mortality rates with most trees dying standing. At least for this region, this pattern seems to be more consistent with climate-induced mortality being the leading cause of death [19,80].

Only stem density coupled with a regional effect were part of the best model describing AGWP while also having a considerable low predictive power (S4 Table and S4 Fig). In fact, although turnover was a good predictor of AGB, no major effect was found in the case of woody productivity (S5 Fig). While at larger scales turnover and AGWP seem to be well correlated (e.g., [24]), in our study we found that carbon dynamics is largely uncoupled from stem dynamics (Table 3 and S5 Fig), and attempting to predict AGWP using forest structure variables in our plots resulted in overall poor correlations and high errors for most of the models tested (S4 Table).

In the relationship between tree mortality and productivity, at least four different mechanisms may be at play, with the dominant mechanisms depending on whether the underlying productivity gradients are caused by climate or soil fertility [81]. In our case, the best models describing AGWP, i.e., the ones with the lowest AICc, seem to confirm that, at least for our plots, woody productivity is largely driven by a combined effect from climate, mostly in the form of water availability (i.e., AET, dry season and CWD), and to a lesser extent by stem density (S4 Table). For instance, in an analysis conducted at the pantropical scale, woody productivity was found to be largely driven by seasonal variation in precipitation and evapotranspiration respectively [82], likely indicating the potential for an overall decrease in tropical forest productivity under a drier climate scenario. Our results show the inherent complexity underlying these patterns and how, for instance, some sites with high mortality rates also have high AGWP (e.g., Western Plains), while the highest productivity was found where turnover rates in general were among the lowest across all regions (e.g., Guiana Shield).

The effects of elevation on turnover, AGWP and AGB is less clear. Stands located at higher elevations can attain high AGB while having low rates of productivity and turnover. While we acknowledge a potential effect of using allometric equations to estimate biomass, which are based on lowland forests, the patterns that were found for these sites still hold when basal area was included as a proxy variable for AGB. Moreover, AGB and other structural parameters (i.e., density and basal area) are aligned with other estimates made for mature tropical montane forests [83], including previous studies where a reduced number of the plots included here (e.g., “Carbonera” cluster) were also used [84]. One potential explanation for these results is that, despite having a clear dry season, lower temperatures at higher elevations promotes lower evapotranspiration and a much higher carbon residence time (Mean of 74.7 years for Andes High elevation forests–S3 Table). Furthermore, this region accounted for the highest stem density and basal area across all plots (Table 2), which may also explain the high AGB values. A similar trend in stem density but not for basal area was found in a tropical Andean gradient [83], but high values in AGB for sites between 2000 and 3000 meters in altitude are common for tropical montane forests [85].

With regards to AGWP, most studies have shown a decrease in net primary productivity with elevation, in most cases as a response of cooler temperatures, fog, reduced light incidence and higher relative humidity [83]. For instance, in an elevation gradient in Amazonia spanning sites from lowland forests up to 3,000 meters in elevation, Doughty et al. [86] found that forests produce biomass less efficiently in stands with residence times > 40 years and in stands with lower soil fertility.

In this study we have tested empirical models that have been applied to other regions in South America, while expanding the analysis to include other forest-types, such as the highly dynamic forests of the Western Plains region, the dry forests in the Caribbean coast, and the high elevation montane forests in the Venezuelan Andes, with the overall aim to contribute to the understanding how the structure of tropical forests influences forest function over a contrasting environmental gradient, and at a scale and region previously unexplored.

Adequately characterizing patterns of turnover, AGB and AGWP over a broad range of environmental conditions in tropical forests presents multiple challenges. For instance, having an adequate and balanced number of sites across all regions and monitoring these simultaneously are ideal, but constitutes an important limitation of this study. Future work should focus on expanding the number of sites and increasing the number of censuses in each region, particularly in the Central Amazon area, to further test the explanatory power of some of our conclusions. Moreover, the use of remote sensing techniques to increase our sample size and to better predict forest structure in many forest types across the tropics (e.g., [87,88]) would further connect the study of tropical forest structure and function at this scale and region.

## Conclusions

Overall, we found that 1) variation in turnover rates in Venezuelan forests are mostly explained by temperature and water availability, combined with stand-level parameters such as basal area and wood density, and that seasonal mature forests in the Western Plains are the most dynamic types; 2) turnover rates and climate are key drivers of forest biomass: where turnover rates are low, mostly as a result of shorter dry seasons (e.g., Central Amazon and Guiana Shield) or low temperatures at higher elevations (e.g., high elevation forests in the Andes), forests tend to have higher AGB with stands dominated by medium to high wood density species; 3) AGWP in Venezuelan forests is largely controlled by the amount of water available, while the effects of stem turnover or forest structural variables is less clear.

Our findings strongly implicate that climate acts as a fundamental driver of neotropical forest turnover, AGB and AGWP. This study therefore has important implications in the context of climate change, given that recent increases in drought frequency have impacted the dynamics of tropical forests by inducing higher rates of tree mortality while diminishing their capacity to store biomass (e.g., [53,89]). If the trend to stronger dry seasons continue, some of the sites included here are likely to be increasingly challenged to continue providing the key ecosystem services of productivity, carbon storage and sequestration.

## Supporting information

S1 Table General description of permanent plots. ^a^ Climatic Water Deficit (CWD) as in Chave et al. 2014 was obtained from a global climate layer for the long-term average of CWD at 2.5 arc-minute resolution. See: http://chave.ups-tlse.fr/pantropical_allometry.htm. It is measured as the difference between rainfall and evapotranspiration during dry months only and is, by definition, negative. Plots with CWD = 0 are not seasonally water-stressed, and sites with very negative CWD values are strongly seasonally water-stressed. ^b^ Seasonality: 0 = Aseasonal (0–1 dry months); 1 = Slightly seasonal (2–3 dry months); 2 = Seasonal (> 3 dry months). ^c^ Soil fertility: 0 = poor nutrient; 1 = richer. This information is derived from literature review and partial soil data available from a reduced number of plots. Shaded cells indicate climatic data obtained directly from local weather stations, while WorldClim database was used for the rest.
(XLSX)

S2 Table Pair-wise thau (τ) correlation between climate, dynamic and structure variables. Green colored cells highlight positive correlations > 0.5. Orange cells are negative correlations < -0.5. Gray cells are positive correlations between 0.2 and 0.5. Blue cells are negative correlations between -0.2 and -0.5.
(DOCX)

S3 Table Estimates of turnover rates, aboveground biomass and productivity for all plots. (DOCX)

S4 Table Parameters of a series of Generalized Least Squares (GLS) models for turnover, aboveground biomass (AGB) and aboveground woody productivity (AGWP) among 50 forest plots in six different regions in Venezuela. Models were fitted upon prior information and region was added as an additional factor with interactions as appropriate (+ symbol). All models incorporated a Gaussian spatial correlation structure to account for spatial autocorrelation. Models are ranked by AICc values, and the final selected model is highlighted in bold.
(XLSX)

S1 Fig Bioclimatic description of 50 plots in Venezuelan forests. (TIFF)

S2 Fig **A) Kendall’s tau correlation matrix for 10 environmental variables used in the principal component analysis; B) Inertia plot of PCA; C) Relationships between three major axes of variation by region**.
(DOCX)

S3 Fig **Boxplots of turnover rates (A-B), AGB (C-D), and AGWP (E-F) by three seasonality conditions, and two major soil fertility groups. Letters indicate results from pos-hoc tests when significant differences were found:** Turnover and seasonality: F = 4.669, p = 0.014*; Turnover and fertility: F = 13.19, p = 0.000682 ***; AGB and seasonality: F = 6.774, p = 0.003**; AGB and fertility: F = 8.933, p = 0.004**; AGWP and seasonality: F = 4.488, p = 0.0165*; AGWP and fertility: F = 1.102, p = 0.299 ns.
(TIFF)

S4 Fig **Relationships between the predicted and estimated values of turnover rates (A-B), AGB (C-D), and AGWP (E-F) based on the “best” regression models selected. Left panel refers to climatic models, while the right panel shows structure-based models for each response variable.** Correlation values here are based on simple linear models between predicted and estimated values. For additional information on all the models tested see S4 Table.
(TIFF)

S5 Fig **Relationships between turnover rates and aboveground biomass (A-C), and aboveground woody productivity (D-F).** Red line indicates the mean of each turnover rate. Shaded line is the confidence interval of the linear fit between pairs of variables.
(TIFF)
