# Supplementary material for: Environmental drivers of forest structure and stem turnover across Venezuelan tropical forests
Source: PLoS One. 2018 Jun 21;13(6):e0198489. doi: 10.1371/journal.pone.0198489 (PMC6013196; doi:10.1371/journal.pone.0198489)
Supplement: S3 Table — (DOCX) [file pone.0198489.s003.docx]

**S3 Table. Estimates of turnover rates, aboveground biomass and productivity for all plots.**

| **Plot Code** | **Forest structure** | | | **Forest demography** | | | **Forest carbon** | | | | | |
| --- | --- | --- | --- | --- | --- | --- | --- | --- | --- | --- | --- | --- |
|  | **Avg Plot  Basal Area (m^2^ ha^-1^)** | **Avg. Plot Stem density (stems ha^-1^)** | **Avg. Plot Wood Density (g cm^-3^)** | **Recruitment interval corrected ^a^ (% year^-1^)** | **Mortality interval corrected ^a^ (% year^-1^)** | **Turnover interval corrected ^a^ (% year^-1^)** | **AGB  (Mg C ha^-1^) ^b^** | **AGB loss (Mg C ha^-1^ year^-1^)** | **AGB growth (Mg C ha^-1^ year^-1^)** | **AGB recruit (Mg C ha^-1^ year^-1^)** | **AGWP (Mg C ha^-1^ year^-1^) ^c^** | **Carbon Residence Time (years) ^d^** |
| ACL-01 | 28.496 | 322 | 0.649 | 1.191 | 1.550 | 1.371 | 176.848 | 2.847 | 3.698 | 0.102 | 3.800 | 46.54 |
| BAC-01 | 26.734 | 346 | 0.500 | 2.204 | 2.831 | 2.518 | 113.500 | 2.489 | 2.615 | 0.387 | 3.002 | 37.81 |
| BAC-02 | 21.214 | 290 | 0.531 | 3.431 | 4.136 | 3.784 | 102.640 | 1.859 | 2.020 | 0.902 | 2.922 | 35.13 |
| BAC-03 | 35.289 | 248 | 0.567 | 2.367 | 2.562 | 2.464 | 206.230 | 1.136 | 2.361 | 0.591 | 2.952 | 69.87 |
| BAC-04 | 28.262 | 302 | 0.599 | 2.243 | 2.745 | 2.494 | 135.579 | 1.331 | 1.722 | 0.189 | 1.911 | 70.95 |
| BAC-05 | 19.401 | 380 | 0.508 | 3.296 | 3.600 | 3.448 | 77.786 | 1.861 | 2.157 | 0.866 | 3.023 | 25.73 |
| BAC-06 | 29.977 | 244 | 0.512 | 2.481 | 2.643 | 2.562 | 150.246 | 3.113 | 3.538 | 0.345 | 3.883 | 38.69 |
| CAI-01 | 22.000 | 310 | 0.547 | 3.174 | 3.841 | 3.507 | 117.096 | 3.155 | 2.820 | 0.430 | 3.250 | 36.03 |
| CAI-02 | 21.594 | 240 | 0.522 | 1.993 | 1.881 | 1.937 | 113.483 | 2.030 | 1.558 | 0.490 | 2.048 | 55.41 |
| CAI-03 | 15.287 | 258 | 0.524 | 3.292 | 3.232 | 3.262 | 80.404 | 1.923 | 1.724 | 0.399 | 2.123 | 37.86 |
| CAI-04 | 22.874 | 248 | 0.535 | 3.421 | 2.979 | 3.200 | 115.821 | 1.568 | 1.878 | 0.444 | 2.322 | 49.88 |
| CAI-05 | 21.744 | 332 | 0.578 | 2.748 | 2.643 | 2.695 | 114.254 | 1.449 | 2.184 | 0.400 | 2.584 | 44.21 |
| CAI-06 | 26.021 | 274 | 0.577 | 1.878 | 1.434 | 1.656 | 150.581 | 0.807 | 2.239 | 0.241 | 2.481 | 60.71 |
| CAT-02 | 18.110 | 314 | 0.542 | 3.029 | 2.671 | 2.850 | 81.251 | 1.972 | 2.656 | 0.228 | 2.884 | 28.18 |
| CAT-03 | 28.964 | 280 | 0.560 | 2.126 | 1.985 | 2.055 | 144.203 | 1.099 | 3.039 | 0.159 | 3.198 | 45.09 |
| CBN-01 | 27.486 | 756 | 0.543 | 1.650 | 1.334 | 1.492 | 136.293 | 1.348 | 1.892 | 0.428 | 2.320 | 58.74 |
| CBN-02 | 27.970 | 926 | 0.538 | 1.388 | 1.187 | 1.288 | 113.619 | 1.524 | 1.661 | 0.419 | 2.080 | 54.62 |
| CBN-03 | 39.309 | 582 | 0.490 | 1.423 | 1.239 | 1.331 | 184.798 | 1.353 | 1.783 | 0.188 | 1.971 | 93.76 |
| CBN-04 | 33.142 | 572 | 0.524 | 1.762 | 2.036 | 1.899 | 179.899 | 2.362 | 1.745 | 0.714 | 2.459 | 73.16 |
| CBN-05 | 32.376 | 774 | 0.587 | 1.445 | 1.756 | 1.601 | 154.846 | 3.072 | 1.314 | 0.236 | 1.550 | 99.88 |
| CBN-06 | 32.308 | 634 | 0.609 | 1.191 | 1.800 | 1.495 | 185.327 | 3.181 | 1.268 | 0.936 | 2.204 | 84.09 |
| CLA-03 | 24.342 | 468 | 0.733 | 1.811 | 1.364 | 1.588 | 125.518 | 0.879 | 1.825 | 0.269 | 2.093 | 59.96 |
| CLA-04 | 24.586 | 640 | 0.759 | 1.702 | 1.839 | 1.770 | 128.517 | 0.966 | 2.032 | 0.350 | 2.383 | 53.94 |
| CRZ-01 | 17.662 | 547.5 | 0.576 | 2.191 | 0.648 | 1.419 | 70.749 | 0.469 | 1.932 | 0.309 | 2.240 | 31.58 |
| ECM-06 | 44.421 | 502 | 0.523 | 0.323 | 1.150 | 0.737 | 204.674 | 1.555 | 2.678 | 0.026 | 2.704 | 75.69 |
| ELD-01 | 28.109 | 430 | 0.738 | 0.903 | 1.399 | 1.151 | 234.506 | 2.323 | 3.724 | 0.121 | 3.845 | 60.99 |
| ELD-02 | 31.171 | 494 | 0.742 | 0.483 | 0.712 | 0.597 | 266.224 | 0.935 | 3.353 | 0.073 | 3.426 | 77.70 |
| ELD-03 | 21.118 | 546 | 0.674 | 2.192 | 1.783 | 1.987 | 136.338 | 4.044 | 3.271 | 0.372 | 3.643 | 37.43 |
| ELD-04 | 27.842 | 672 | 0.645 | 1.048 | 1.612 | 1.330 | 156.565 | 3.592 | 4.145 | 0.201 | 4.346 | 36.03 |
| EMC-02 | 26.058 | 197 | 0.621 | 3.538 | 1.730 | 2.634 | 151.633 | 0.503 | 2.481 | 1.683 | 4.165 | 36.41 |
| ESM-05 | 42.872 | 532 | 0.589 | 1.427 | 1.980 | 1.703 | 205.316 | 2.494 | 3.558 | 0.210 | 3.768 | 54.48 |
| GUR-05 | 16.843 | 328 | 0.775 | 1.346 | 1.074 | 1.210 | 133.051 | 1.273 | 2.119 | 0.260 | 2.379 | 55.94 |
| GUR-06 | 21.306 | 410 | 0.764 | 2.343 | 1.543 | 1.943 | 174.418 | 4.488 | 1.904 | 0.278 | 2.182 | 79.95 |
| HCU-01 | 36.146 | 452 | 0.660 | 0.405 | 0.728 | 0.567 | 237.270 | 0.808 | 2.715 | 0.043 | 2.758 | 86.03 |
| HSP-01 | 19.412 | 348 | 0.580 | 2.610 | 1.832 | 2.221 | 96.122 | 1.062 | 1.871 | 0.313 | 2.184 | 44.01 |
| MOL-04 | 31.883 | 654 | 0.557 | 1.323 | 1.441 | 1.382 | 159.029 | 1.914 | 2.578 | 0.179 | 2.757 | 57.69 |
| RIO-01 | 29.389 | 536 | 0.708 | 1.008 | 1.074 | 1.041 | 226.454 | 3.116 | 3.541 | 0.145 | 3.687 | 61.43 |
| RIO-02 | 30.773 | 580 | 0.700 | 0.974 | 1.540 | 1.257 | 225.897 | 3.416 | 3.380 | 0.150 | 3.530 | 64.00 |
| SAR-02 | 9.213 | 338 | 0.731 | 2.293 | 2.380 | 2.337 | 47.660 | 0.528 | 0.591 | 0.236 | 0.827 | 57.65 |
| SCR-04 | 29.085 | 734 | 0.673 | 1.887 | 1.265 | 1.576 | 183.288 | 1.801 | 1.756 | 0.580 | 2.337 | 78.43 |
| SCR-05 | 28.288 | 579.5 | 0.685 | 2.941 | 2.051 | 2.496 | 201.301 | 3.455 | 2.360 | 0.799 | 3.160 | 63.71 |
| SDL-01 | 31.029 | 657.5 | 0.719 | 0.350 | 1.481 | 0.915 | 237.089 | 5.692 | 3.812 | 0.052 | 3.865 | 61.34 |
| SDL-04 | 34.288 | 1065 | 0.628 | 1.605 | 2.650 | 2.127 | 204.805 | 2.532 | 3.498 | 0.279 | 3.777 | 54.23 |
| SDL-05 | 41.634 | 1165 | 0.560 | 0.845 | 1.996 | 1.421 | 257.185 | 6.765 | 1.166 | 0.100 | 1.266 | 203.12 |
| SEU-01 | 36.741 | 926 | 0.555 | 2.260 | 1.528 | 1.894 | 173.818 | 1.859 | 2.086 | 0.501 | 2.587 | 67.20 |
| SEU-02 | 34.279 | 1135 | 0.591 | 1.978 | 1.348 | 1.663 | 140.132 | 1.652 | 1.700 | 0.612 | 2.312 | 60.61 |
| SEU-03 | 41.203 | 638 | 0.645 | 1.694 | 1.395 | 1.544 | 224.870 | 2.315 | 2.373 | 0.281 | 2.654 | 84.73 |
| SEU-04 | 38.923 | 652 | 0.614 | 2.370 | 1.856 | 2.113 | 190.334 | 2.998 | 2.431 | 0.405 | 2.836 | 67.11 |
| SEU-05 | 37.852 | 866 | 0.654 | 1.851 | 1.671 | 1.761 | 170.767 | 2.770 | 1.586 | 0.466 | 2.052 | 83.24 |
| SEU-06 | 37.599 | 746 | 0.650 | 2.231 | 1.801 | 2.016 | 171.071 | 2.331 | 1.495 | 0.420 | 1.915 | 89.32 |

^a^ Rates are calculated and corrected for every census interval, and then weighted by census length. For details on the correction approach see methods section.

^b^ Total aboveground biomass based on plot inventory data (trees > 10 cm) + 6.2% from small trees (< 10 cm D) as in Malhi et al. (2006).

^c^ AGWP = AGB growth + AGB recruit.

^d^ Carbon residence time = AGB / AGWP.
