# Supplementary figures and images for: Environmental drivers of forest structure and stem turnover across Venezuelan tropical forests
Source: PLoS One. 2018 Jun 21;13(6):e0198489. doi: 10.1371/journal.pone.0198489 (PMC6013196; doi:10.1371/journal.pone.0198489)

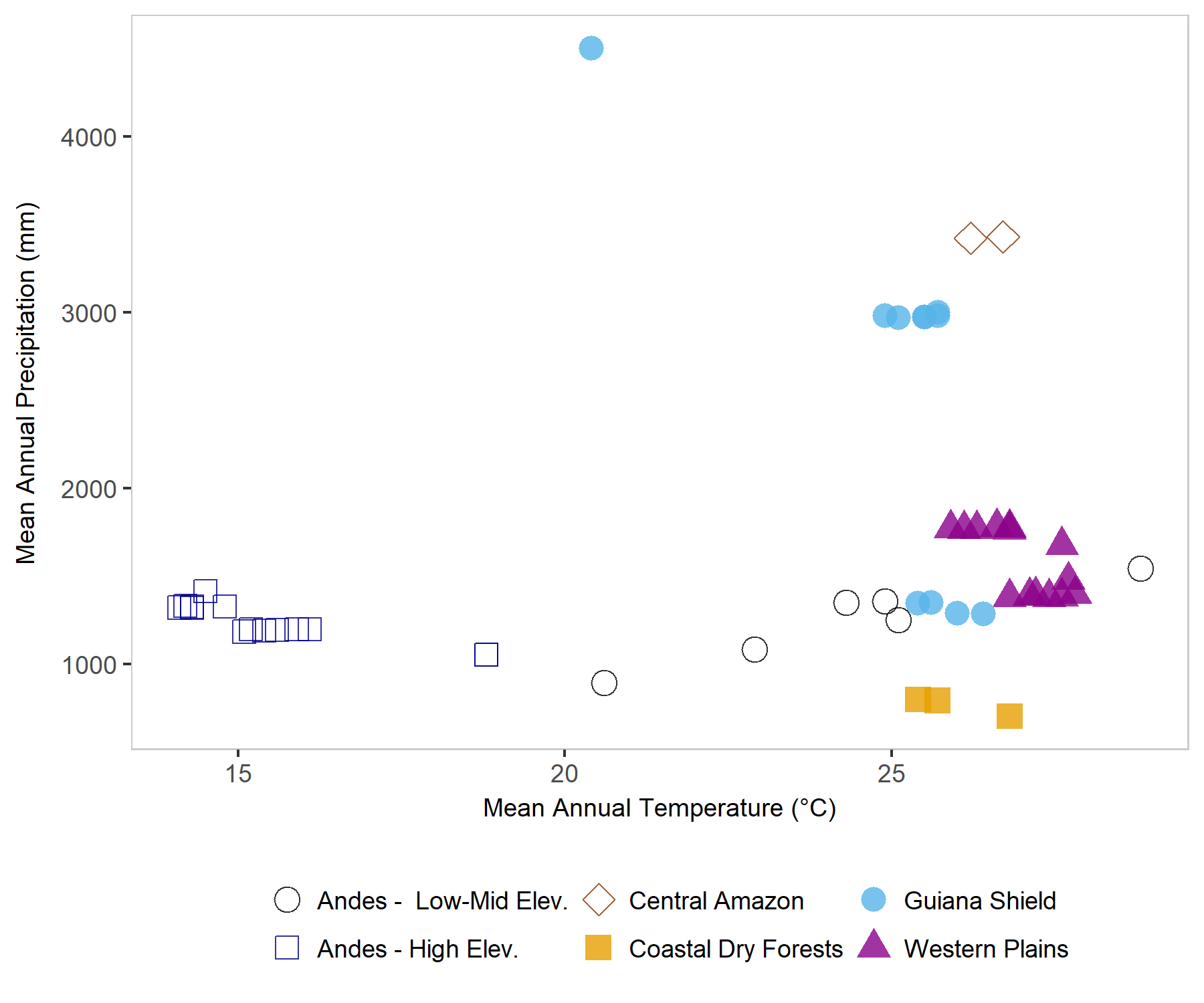

Supplement: S1 Fig — (TIFF) [file pone.0198489.s005.tiff]

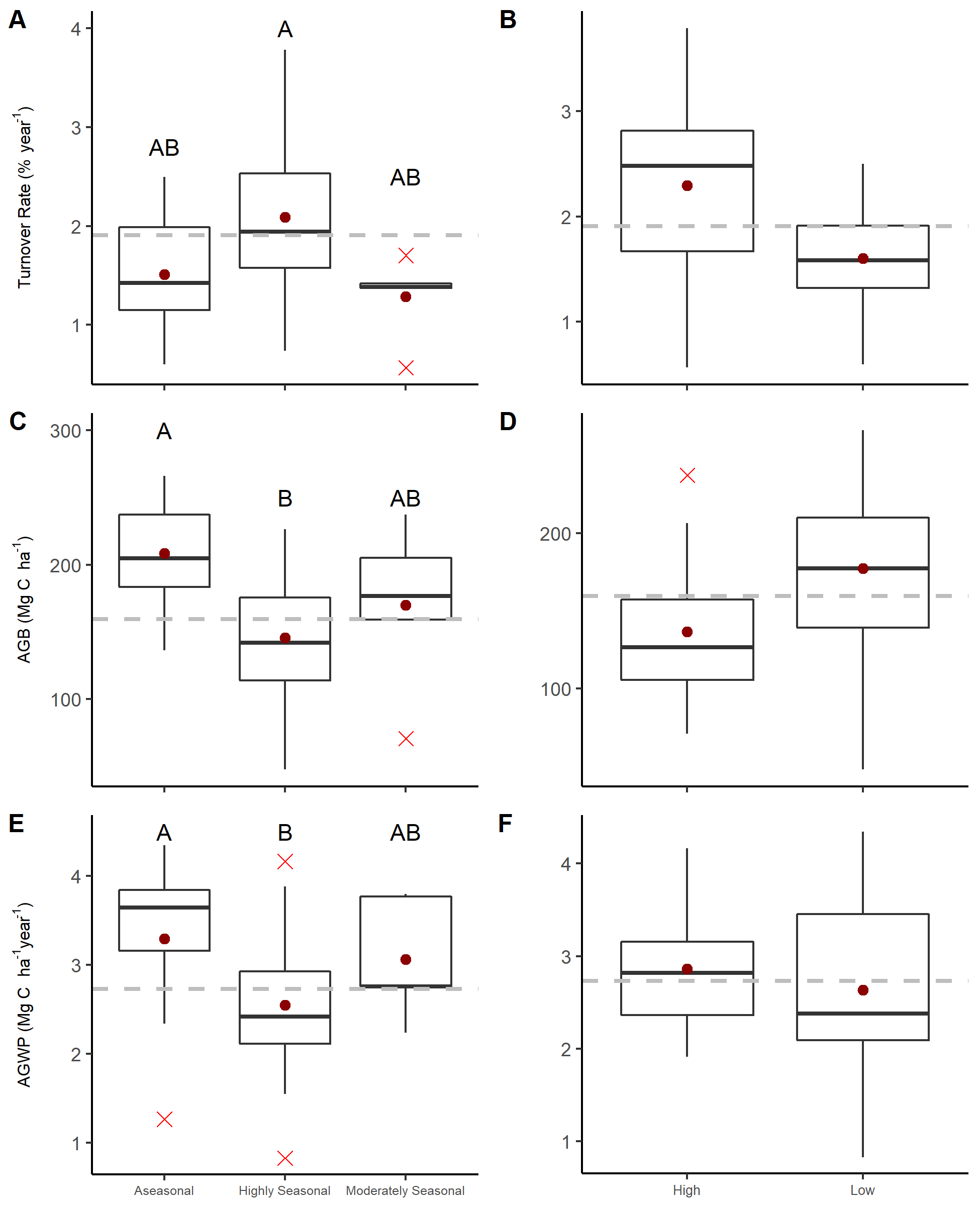

Supplement: S3 Fig — Boxplots of turnover rates (A-B), AGB (C-D), and AGWP (E-F) by three seasonality conditions, and two major soil fertility groups. Letters indicate results from pos-hoc tests when significant differences were found: Turnover and seasonality: F = 4.669, p = 0.014*; Turnover and fertility: F = 13.19, p = 0.000682 ***; AGB and seasonality: F = 6.774, p = 0.003**; AGB and fertility: F = 8.933, p = 0.004**; AGWP and seasonality: F = 4.488, p = 0.0165*; AGWP and fertility: F = 1.102, p = 0.299 ns. (TIFF) [file pone.0198489.s007.tiff]

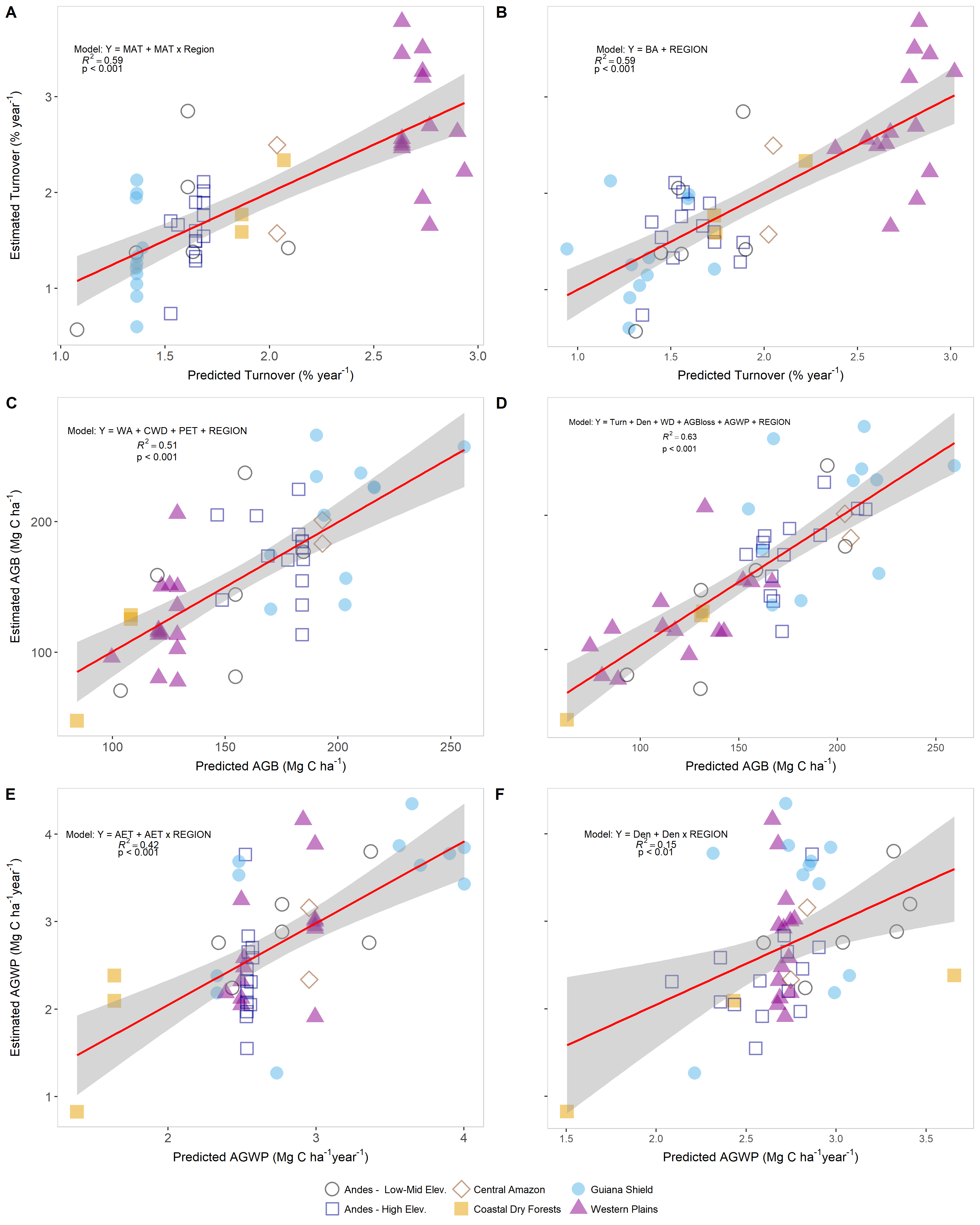

Supplement: S4 Fig — Relationships between the predicted and estimated values of turnover rates (A-B), AGB (C-D), and AGWP (E-F) based on the “best” regression models selected. Left panel refers to climatic models, while the right panel shows structure-based models for each response variable. Correlation values here are based on simple linear models between predicted and estimated values. For additional information on all the models tested see S4 Table. (TIFF) [file pone.0198489.s008.tiff]

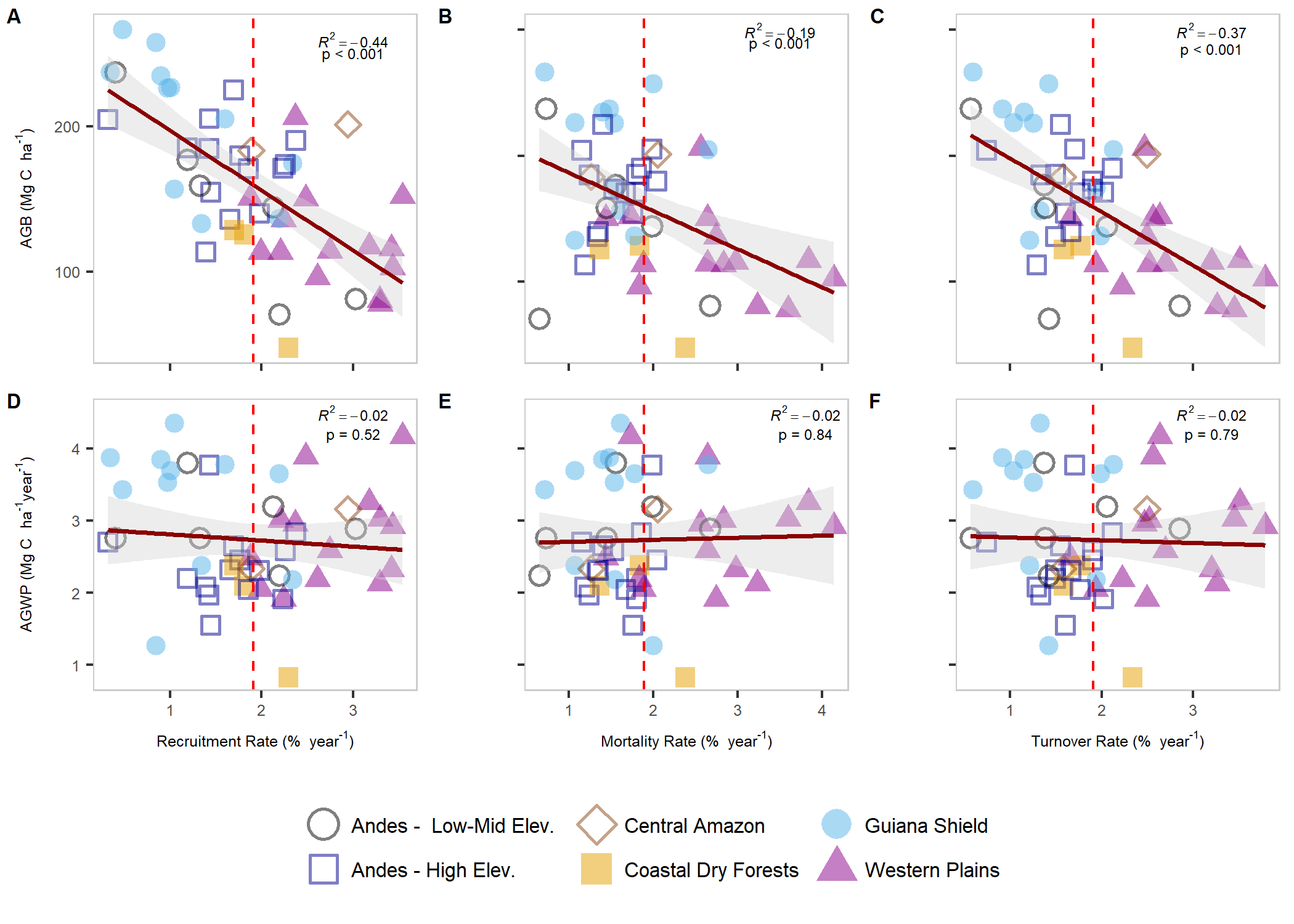

Supplement: S5 Fig — Relationships between turnover rates and aboveground biomass (A-C), and aboveground woody productivity (D-F). Red line indicates the mean of each turnover rate. Shaded line is the confidence interval of the linear fit between pairs of variables. (TIFF) [file pone.0198489.s009.tiff]
