# Supplementary material for: Environmental drivers of forest structure and stem turnover across Venezuelan tropical forests
Source: PLoS One. 2018 Jun 21;13(6):e0198489. doi: 10.1371/journal.pone.0198489 (PMC6013196; doi:10.1371/journal.pone.0198489)
Supplement: S2 Fig — A) Kendall’s tau correlation matrix for 10 environmental variables used in the principal component analysis; B) Inertia plot of PCA; C) Relationships between three major axes of variation by region. (DOCX) [file pone.0198489.s006.docx]

**A)**


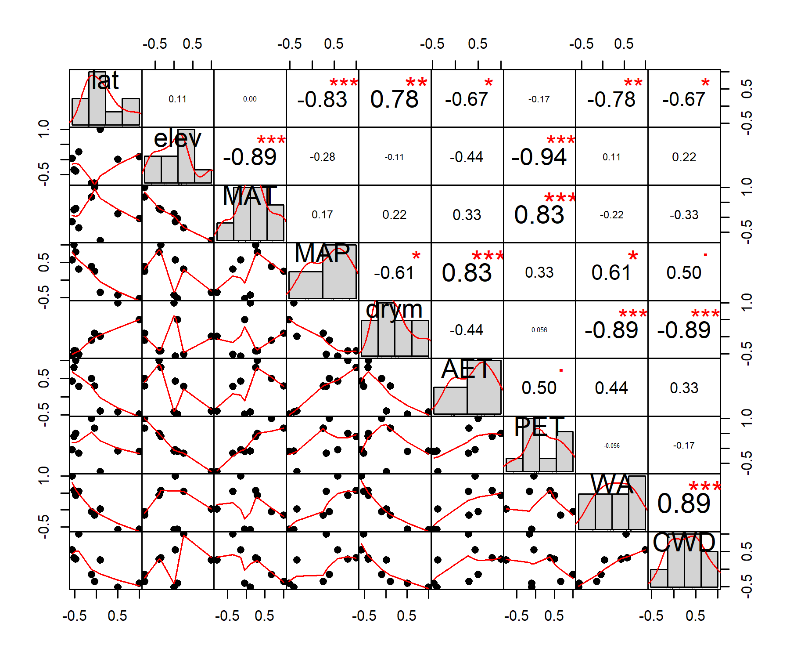


**B)**


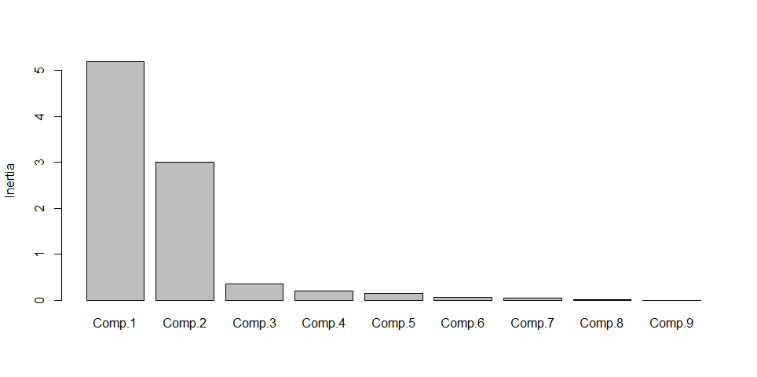


**C)**


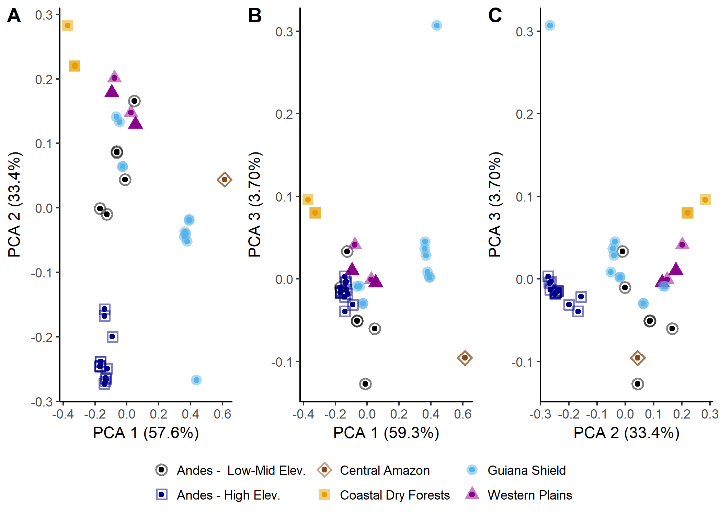


**S2 Fig. A) Kendall’s tau correlation matrix for 10 environmental variables used in the Principal Component Analysis; B) Inertia plot of PCA; C) Relationships between three major axes of variation by region.**
